# Clinical drug interactions between voriconazole and 38 other drugs: a retrospective analysis of adverse events

**DOI:** 10.3389/fphar.2024.1292163

**Published:** 2024-09-30

**Authors:** Ben-Nian Huo, Ling Shu, Jian-Wen Xiao, Nan-Ge Yin, Mao-Lin Ai, Yun-Tao Jia, Lin Song

**Affiliations:** ^1^ Department of Pharmacy, Children’s Hospital of Chongqing Medical University, National Clinical Research Center for Child Health and Disorders, Ministry of Education Key Laboratory of Child Development and Disorders, Chongqing Key Laboratory of Pediatric Metabolism and Inflammatory Diseases, Chongqing, China; ^2^ Department of Pharmacy, People’s Hospital of Chongqing Liangjiang New Area, Chongqing, China; ^3^ Department of hematology, Children’s Hospital of Chongqing Medical University, Chongqing, China

**Keywords:** voriconazole, PPIs, NSAIDs, immunosuppressants, drug–drug interaction, safety, FAERS

## Abstract

**Background:**

Voriconazole (VRZ) is involved in a variety of drug‒drug interactions (DDIs), but few studies have reported adverse events (AEs) associated with the DDIs of VRZ. The primary goal of this study was to analyse the potential risk factors for AEs caused by DDIs between VRZ and other drugs via the OpenVigil FDA platform and to provide a reference for preventing VRZ DDIs and monitoring clinically related adverse drug events.

**Methods:**

A retrospective pharmacovigilance study was conducted to investigate the AEs related to DDIs between VRZ and four categories of drugs: proton pump inhibitors (PPIs), non-steroidal anti-inflammatory drugs (NSAIDs), immunosuppressants, and other antibacterial drugs. AE information for the target drugs from the first quarter of 2004 to the third quarter of 2022 was downloaded from the OpenVigil FDA data platform. Four frequency statistical models—the reporting ratio method, Ω shrinkage measure model, combination risk ratio model, and the chi-square statistics model—were used to analyse the AEs related to DDIs and evaluate the correlation and influence of sex and age between the drug(s) and the target AEs detected.

**Results:**

A total of 38 drugs were included, with 262 AEs detected by at least one of the four models and 48 AEs detected by all four models. Some 77 detected AEs were significantly positively correlated with DDIs and were related to higher reporting rates of AEs than when used alone. Graft-versus-host disease was the AE that had the strongest correlation with the drug interaction between VRZ and immunosuppressants (tacrolimus, mycophenolate mofetil, cyclophosphamide, and cyclosporine), and multiple organ dysfunction syndrome was correlated with VRZ in combination with other antibacterial drugs (linezolid, meropenem, cefepime, and vancomycin). Significant sex and age differences in the target AEs were detected for five and nine target drugs, respectively. For VRZ in combination with linezolid, aggravated conditions and respiratory failure should be given more attention in male patients, and mycophenolate mofetil and respiratory failure in female patients. When conditions are aggravated, febrile neutropenia and septic shock should be of particular concern in patients over 18 years of age who use VRZ in combination with ceftazidime, ciprofloxacin, or cytarabine. In patients aged under 18, septic shock should be considered when VRZ is used in combination with meropenem and dexamethasone.

**Conclusion:**

AEs related to DDIs should receive more attention when VRZ is used in combination with PPIs (renal impairment), NSAIDs (constipation and renal failure), immunosuppressants (graft versus host disease, septic shock) and other antibacterial drugs (multiple organ dysfunction syndrome, febrile neutropenia, and respiratory failure). Considering the influence of sex and age differences in VRZ DDIs, these factors need to be considered when assessing the risk of AEs in patients receiving VRZ and other drugs.

## 1 Background

Voriconazole (VRZ) is a synthetic second-generation broad-spectrum triazole agent that is recommended for first-line treatment and the prevention of a variety of invasive fungal diseases such as invasive aspergillosis, oesophageal candidiasis, and severe infections caused by *Scedosporium apiospermum* and *Fusarium* spp. (Lee et al., 2021; Pfizer, 2020). VRE is highly prone to drug‒drug interactions (DDIs), mainly related to cytochrome P450 (CYP450) enzymes, as many commonly used prescription drugs are also metabolized through these enzymes (Job et al., 2016). Many additional factors, such as plasma concentration, age, and other complications, may also affect the DDI of VRZ (Pfizer, 2020; Tian et al., 2021). Therefore, health professionals should be more cautious when prescribing VRZ because of the high risk of DDIs.

A number of studies have shown that DDIs are the primary cause of adverse events (AEs) and are considered one of the most serious global health concerns (Kantor et al., 2015). In clinical practice, DDIs often occur in patients with complications and concomitant medications (Prybys et al., 2002). Previous studies have reported that with increasing rates of concomitant medications, the risk of AEs increased from 13% for two drugs to 58% for five drugs (Kantor et al., 2015; Prybys et al., 2002). Rodrigues and Oliveira (2016) reported that the majority of fungal infection patients suffer from other diseases and need to be treated with other drugs, which is more likely to lead to DDIs. The guidelines recommend that the efficacy and safety of VRZ should be closely monitored when it is used in combination with proton pump inhibitors (PPIs) (e.g., omeprazole, aesomeprazole, pantoprazole, rabeprazole, and lansoprazole), immunosuppressant agents (e.g., glucocorticoids, cyclosporine, and tacrolimus), and other antibacterial drugs (e.g., erythromycin, azithromycin, and clarithromycin) which may lead to unexpected toxicity or decreased therapeutic efficacy due to potential DDIs (Chen et al., 2018). Therefore, identifying potential DDIs between VRZ and commonly prescribed drugs is of great clinical importance and may help to reduce the risk of AEs.

Existing research has revealed a variety of DDIs between VRZ and other drugs, but the occurrence of interaction-related AEs of VRZ is rarely reported (Groll et al., 2017). A publicly available FDA AE Reporting System (FAERS) database was used as an efficient tool for identifying DDIs of VRZ. The US FDA Open Data Project (OpenVigil FDA) platform is a novel web-based pharmacovigilance analysis tool that uses the OpenFDA online interface to access the drug-event dataset from FAERS; it can provide disproportionality analyses to estimate rare or new adverse drug reactions and check arbitrary combinations of two drugs for unknown DDI signals (Meng et al., 2022; Böhm et al., 2016; Böhm et al., 2021). Thus, the primary goal of this study was to analyse the potential risk factors for AEs caused by DDIs between VRZ and the drugs usually used in combination via the OpenVigil FDA platform and to provide a reference for preventing VRZ DDIs and monitoring clinically related adverse drug events.

## 2 Methods

### 2.1 Study design

A cross-sectional and retrospective pharmacovigilance study was conducted to investigate AEs related to drug interactions between VRZ and other drugs usually used in combination with VRZ and to search for possible DDIs. On the basis of the relevant literature and guidelines (Job et al., 2016; Chen et al., 2018; Thomas et al., 2009; Dong et al., 2024; Idoate et al., 2019; Kim et al., 2010), the current situation of the concomitant use of VRZ, and discussion with clinical experts, the four most concerning and widely prescribed drug classes that may affect drug safety and are commonly used in combination with VRE in the clinic—PPIs, non-steroidal anti-inflammatory drugs (NSAIDs), immunosuppressants, and other antibacterial drugs—were included in this analysis.

### 2.2 Data sources and selection criteria

Relevant data from the first quarter of 2004 to the third quarter of 2022 were downloaded from the OpenVigil FDA data platform (https://openvigil.pharmacology.uni-kiel.de/openvigilfda.php). If there were more than three reports of AEs related to VRZ and the target drug interaction (Noguchi and Teramachi, 2020), the AE information of the target drug used alone or in combination with VRZ was further extracted for AE interaction analysis. AEs recorded in the OpenVigil FDA database were coded according to the preferred term (PT) by the International Harmonized Conference on Human Drug Registration Technology (ICH) in the Medical Dictionary for Regulatory Activities Terminology (MedDRA) (Wong et al., 2015).

### 2.3 Statistical analysis

Descriptive analyses were used to summarize the data of the AE reports collected from the OpenVigil FDA platform, and the number (%) was used for qualitative variables. A *P* value of less than 0.05 was considered statistically significant, and all the statistical analyses were performed on a personal computer with the SPSS for Windows statistical package (version 22.0). Image processing for correlation analysis and influencing factor analysis of DDIs was performed with R 4.2.2 software.

#### 2.3.1 Statistical models and criteria for the detection of adverse events

Four frequency statistical models of signal mining were used to calculate the threshold and detect potential AEs related to DDIs (Noguchi and Teramachi, 2020): the reporting ratio method (Böhm et al., 2021), Ω shrinkage measure model (Norén et al., 2008), combination risk ratio model (Susuta and Takahashi, 2014), and the chi-square statistics model (Gosho et al., 2017). The related parameters and algorithms of these models are shown in Supplementary Tables S1–3, and a brief description is provided below.

(1) Reporting ratio method: this method was used to compare the frequency of AEs between drugs used alone and those used in combination. If the observed rate of AEs for the combination was greater than the expected rate (the sum of the occurrence frequency when each drug was used separately) —that is, if the percentage difference (R_diff) was <0—positive safety signals for the DDI of the two drugs were detected.
(2) Ω shrinkage measurement model: this model calculates the logarithm of the ratio between the actual observation value and the expected value of the target AE reports when the drug is used in combination. When Ω > 0.25, positive safety signals for the DDI of the two drugs were detected.
(3) Combination risk ratio model: this model evaluates probabilities of drug interactions by calculating the comprehensive risk of the target AE—that is, to evaluate the ratio of the proportional reporting ratio (PRR) —when two drugs are used together (PRR) and the ratio of the PRR when one of the two drugs is used alone (PRR_1_, PRR_2_). In cases where both drugs were used and the target AE occurred ≥3, a combination risk ratio >2, PRR>2, and χ^2^ > 4, positive safety signals for the DDI of the two drugs were detected (Böhm et al., 2016).
(4) Chi-square statistics model: this model uses chi-square statistics to estimate the discrepancy between the observed and expected values of target AEs with two drugs used together. When χ > 2, the positive safety signals for the DDI of the two drugs were detected.

#### 2.3.2 Correlation analysis

The relative reporting ratio (RRR) was used to quantitatively measure the correlation between the drug(s) and the ^target^ AEs, with positive signals of DDIs detected by the above models; the greater the RRR, the greater the correlation between the drug and the target AE (Glotzbecker et al., 2012). The RRR was calculated as follows:

$$\text{RRR}=\frac{a\times n}{\left(a+b\right)E},$$

where a and b represent the same parameters as in the combination risk ratio model, and the related parameters are shown in Supplementary Table S1. Delta_RRR is the difference between the RRR_E_ value when two drugs are used together and the mean RRR_1_ and RRR_2_ when two drugs are used alone. The Delta_RRR was calculated as follows:

$$\text{Delta}\_\text{RRR}={\text{RRR}}_{E}-\frac{{\text{RRR}}_{1}+{\text{RRR}}_{2}}{2}.$$

The ratio of Delta_RRR to the mean Delta_RRR of all AEs (Delta_RRR_all_), defined as RRR_diff, was used to evaluate the difference in the correlation degree of AEs between two drugs used together or alone (Böhm et al., 2016). The RRR_diff was calculated as follows:

$$\text{RRR}\_\text{diff}=\frac{\text{Delta}\_\text{RRR}}{\text{mean}\left(\;\text{Delta}\_{\text{RRR}}_{\text{all}}\right)}\times 100\%.$$

The RRR_diff values of related adverse drug events with interaction signals detected by the above four models were calculated to evaluate the degree of correlation difference. When RRR_diff >0.75, the correlation between the target AE and the combined use of two drugs is greater than that of two single drugs, which indicates a significant positive correlation and leads to an increased risk of target AEs when the drug is used in combination (Böhm et al., 2016). When RRR_diff < −0.75, the correlation between the target AE and the two drugs used alone is greater than when two drugs are used together, which indicates a significant negative correlation, and the risk of target AEs is greater when two drugs are used alone.

#### 2.3.3 Influencing factors analysis

The DDIs related to adverse drug events with positive interaction signals detected by at least one of the above four models and an RRR_diff value >0.75 were included to evaluate the influence of sex and age. A two-by-two contingency table was used to calculate the reporting odds ratio (ROR) values and 95% confidence intervals (CIs). Related parameters are shown in Supplementary Table S4, and the algorithms were as follows:

$$ROR=\frac{a\times d}{b\times c}95\%CI=e^{In\left(\text{ROR}\right)\pm 1.96\sqrt{\frac{1}{a}+\frac{1}{b}+\frac{1}{c}+\frac{1}{d}.}}$$

Criteria for inclusion in the statistical analysis were a > 5, c > 5, a+c>50. Pearson’s chi-square test was used to compare the differences in the risk of target AEs between female and male patients and between ≤18- and >18-year-old patients. The standards for statistical significance were as follows: a Pearson’s chi-square test with *P* < 0.05 (−log_10_ *P* value >1.301) and a log_2_ROR > 1 indicates that female or ≤18-year-old patients are more likely to have a greater risk of target AEs, and *P* < 0.05 (−log_10_ *P* value > 1.301) and a log_2_ROR < −1 indicate that male or >18-year-old patients tend to have a greater risk of target AEs (Yu et al., 2016).

## 3 Results

### 3.1 General results

There were no AE reports about piperacillin sodium, sulbactam sodium, cefoperazone sodium, sulbactam sodium, cefathiamidine, cefotiam, or ceftizoxime in combination with VRZ recorded in the OpenVigil FDA platform. The z number of AE reports about amoxicillin, clavulanate potassium, cefadroxil, cefaclor, and cefixime combined with VRZ was less than three (Böhm et al., 2016). Thus, 38 drugs commonly used in combination with VRZ were included in this study for the detection of AEs related to DDIs. The 38 are:

(1) PPIs: aesomeprazole, lansoprazole, omeprazole, pantoprazole, and rabeprazole.
(2) NSAIDs: acetaminophen, aspirin, celecoxib, and ibuprofen.
(3) Immunosuppressants: azathioprine, cytarabine, cyclosporine, cyclophosphamide, dexamethasone, hydrocortisone, methylprednisolone, methotrexate, mycophenolate mofetil, tacrolimus, and tocilizumab.
(4) Other antibacterial drugs: amphotericin B, azithromycin, caspofungin, cefazolin, cefepime, cefpodoxime proxetil, ceftazidime, ceftriaxone, cefuroxime, clarithromycin, ciprofloxacin, imipenem, imipenem and cilastatin sodium, levofloxacin, linezolid, meropenem, sulfamethoxazole, and vancomycin.

**FIGURE 1:**
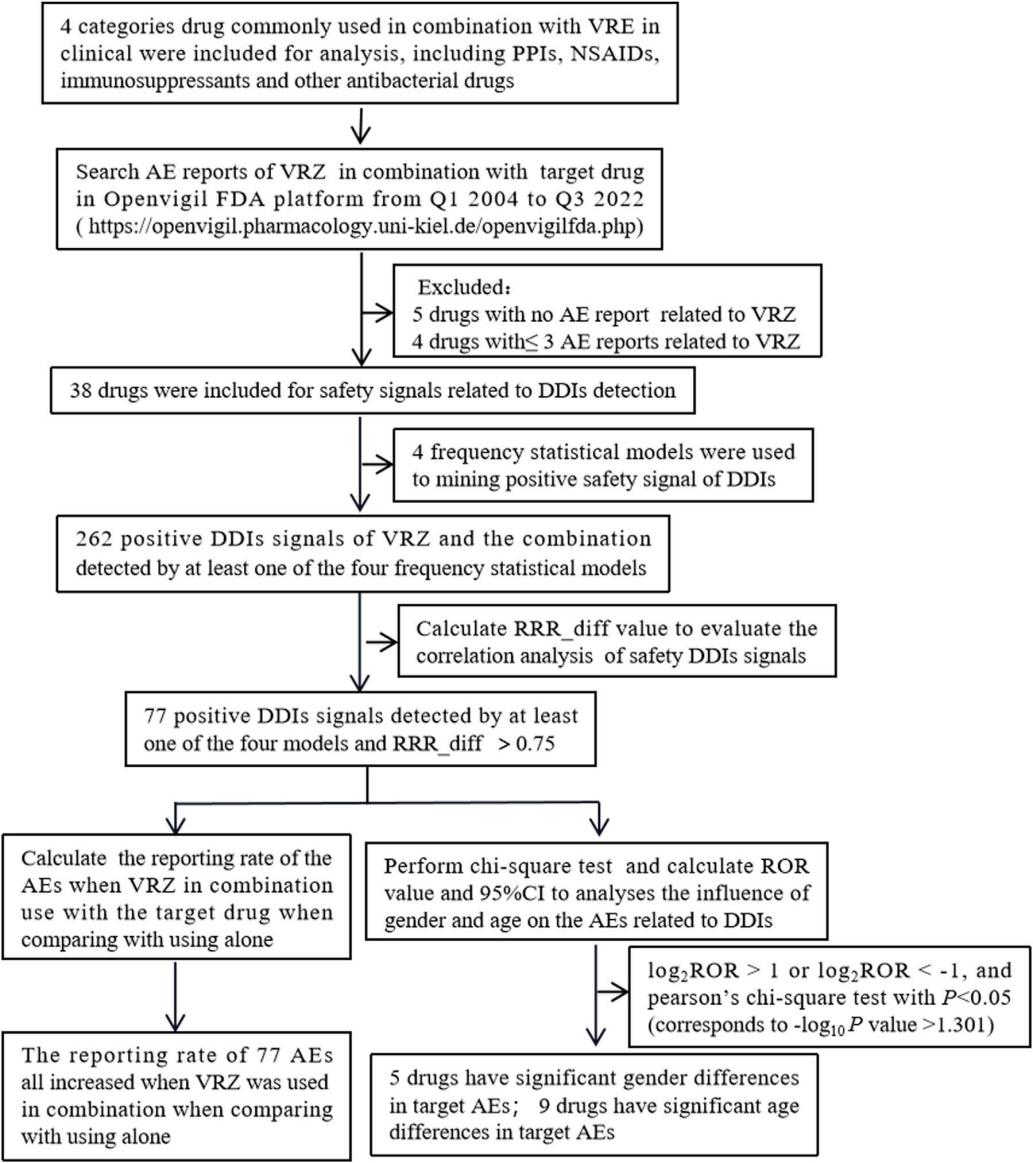
Flow chart of the inclusion of adverse events (Abbreviations: AE, adverse event; DDIs, drug‒drug interactions; RRR, relative reporting ratio; VRZ, voriconazole).

A total of 15,234,431 AE reports were recorded in the FAERS database on the OpenVigil FDA platform as of 30 September 2022, of which 4,563, 2,392, 20,964, and 25,583 AE reports were recorded when VRE was used in combination with PPIs, NSAIDs, immunosuppressants, and other antibacterial drugs, respectively. The flow chart is shown in Figure 1.

### 3.2 VRZ adverse events related to DDIs

The numbers of AE types and positive DDI AEs detected by at least one of the four frequency statistical models when VRZ was used in combination with the 38 drugs and the sex and age distributions of the related patients are presented in Table 1. A total of 262 AEs were detected by at least one of the four frequency statistical models when VRZ and 38 drugs were co-administered. For a specific positive signal result, the more models that detected it, the more reliable the result. Among the 262 AEs, 48 were detected by all four models (Table 2). The most common AEs related to DDIs were anaemia (10.4% (5/48)) and condition aggravated (10.4% (5/48)), followed by constipation (8.3% (4/48)). The results of the other 214 positive AEs related to DDIs of VRZ and the target drug detected by at least one of the above four models are shown in Supplementary Table S5.

**TABLE 1:** Number of AE categories, positive safety signals and the gender, age distribution of the AEs when VRZ used in combination with target drugs^a^.

VRZ + concomitant drug	Number of AE reports^b^	Number of AE categories	Number of positive DDI signals^c^	Number of female	Number of male	Number of ≤18 years	Number of >18 years
VRZ + PPIs	4,563	209	40	2066	2,506	1,201	3,362
Aesomeprazole	610	37	7	243	408	90	520
Lansoprazole	1,106	44	18	612	505	284	822
Omeprazole	1,380	43	5	572	732	368	1,012
Pantoprazole	1,370	44	4	597	801	429	941
Rabeprazole	97	41	6	42	60	30	67
VRZ + NSAIDs	2,392	167	20	1,009	1,479	708	1,684
Acetaminophen	1,507	41	6	697	941	476	1,031
Aspirin	627	47	4	194	399	135	492
Celecoxib	70	38	4	26	38	12	58
Ibuprofen	188	41	6	92	101	85	103
VRZ + Immunosuppressants	20,964	551	67	7,408	12,485	7,007	13,957
Azathioprine	288	42	6	107	206	39	249
Cytarabine	2,556	61	6	912	1,469	1,006	1,550
Cyclosporine	1,432	53	8	541	792	555	877
Cyclophosphamide	1976	57	5	725	1,049	826	1,150
Dexamethasone	2,341	60	6	1,035	1,119	697	1,644
Hydrocortisone	1,348	44	7	549	833	532	816
Methylprednisolone	2,270	51	7	752	1,494	576	1,694
Methotrexate	1,131	38	7	334	888	664	467
Mycophenolate mofetil	2,771	57	5	884	1,682	753	2018
Tacrolimus	4,720	57	7	1,537	2,825	1,307	3,413
Tocilizumab	131	31	3	32	128	52	79
VRZ + other antibacterial drugs	25,583	1,036	135	9,420	15,467	7,936	17,647
Amphotericin B	5,062	71	9	1,664	3,130	1750	3,312
Azithromycin	544	42	3	235	328	213	331
Caspofungin	1,219	62	19	508	747	551	668
Cefazolin	148	51	7	49	95	27	121
Cefepime	1,514	62	6	631	840	490	1,024
Cefpodoxime proxetil	38	49	2	23	21	2	36
Ceftazidime	782	64	5	283	564	155	627
Ceftriaxone	585	59	5	189	353	108	477
Cefuroxime	51	44	2	10	41	17	34
Clarithromycin	334	56	10	99	260	90	244
Ciprofloxacin	2016	51	8	733	1,180	542	1,474
Imipenem	258	61	11	118	185	78	180
Imipenem and cilastatin sodium	258	61	11	81	168	78	180
Levofloxacin	1728	56	5	616	915	586	1,142
Linezolid	1773	68	15	885	923	297	1,476
Meropenem	3,237	64	7	1,157	1976	854	2,383
Sulfamethoxazole	2082	56	3	721	1,302	844	1,238
Vancomycin	3,954	59	7	1,418	2,439	1,254	2,700
Total	53,502	1963	262	19,903	31,937	16,852	36,650

Abbreviation: AE, adverse event; DDIs: drug–drug interactions; NSAIDs, non-steroidal anti-inflammatory drugs; PPIs, proton pump inhibitors; VRZ: voriconazole.

^a^ Due to technical limitations and constant changes of the OpenVigil FDA API, the imbalance of the data extracted from the database was about 7% in this study.

^b^ Reported number of target AEs when two drugs are used together, and its meaning equivalent to D_E_, shown in Supplementary Table 1.

^c^ Positive DDI signals detected by at least one of the four frequency statistical models when VRZ is used in combination with target drugs.

**TABLE 2:** Positive safety signals related to DDIs detected by all four frequency statistical models when VRZ in combined use with target drugs (n = 48)^a^.

Drug	Adverse event (PT)	AE reports (N)^b^	Reporting rate (%)			Reporting ratio method (R_diff)^c^	Ω shrinkage measure model (Ω_0.25_)^d^	Combination risk ratio model CRR (PRR, χ2)	Chi-squared statistics model (χ)
			VRZ	Target drug	Both drugs				
VRZ + Proton pump inhibitors (PPIs)									
Aesomeprazole	Confusional state	19	1.7	1.1	2.8	−3.7	0.4	3.8 (2.4, 53.4)	2.6
Aesomeprazole	Erythema	16	1.0	0.9	2.4	−22.4	0.3	2.6 (3.5, 20.8)	2.2
Aesomeprazole	Hemoglobin decreased	13	0.9	0.8	1.9	−15.2	0.7	4.1 (3.2, 39.9)	3.0
Lansoprazole	Anemia	48	1.7	1.7	6.8	−49.9	0.7	4.5 (3.6, 181.5)	5.2
Lansoprazole	Platelet count decreased	29	1.7	0.8	4.1	−39.8	0.2	5.1 (2.2, 129.1)	2.1
Lansoprazole	Decreased appetite	38	1.0	1.5	5.3	−53.7	0.8	3.2 (3.4, 78.7)	5.2
Lansoprazole	Constipation	34	0.7	1.6	4.8	−52.0	0.5	3.1 (2.8, 66.0)	3.8
Omeprazole	Confusional state	47	1.6	1.5	3.9	−20.8	0.4	4.1 (3.0, 154.7)	3.7
Omeprazole	Thrombocytopenia	34	1.6	0.8	2.8	−16.3	0.2	4.6 (2.2, 129.8)	2.3
Omeprazole	Vision blurred	25	1.2	0.9	2.1	−0.3	0.2	2.7 (2.2, 37.0)	2.1
Omeprazole	Hemoglobin decreased	24	0.8	1.0	2.0	−7.9	0.2	3.3 (2.5, 52.1)	2.0
Pantoprazole	Thrombocytopenia	47	1.5	1.2	3.5	−23.7	0.4	6.4 (3.2, 291.4)	3.8
Rabeprazole	Renal impairment	7	1.5	1.2	5.2	−49.3	0.9	18.2 (6.4, 138.8)	3.2
Rabeprazole	Constipation	6	0.8	1.9	4.4	−39.4	0.7	6.2 (5.8, 31.1)	2.7
Rabeprazole	Insomnia	7	0.7	2.2	5.2	−43.1	0.8	5.3 (5.8, 30.0)	3.0
VRZ + non-steroidal anti-inflammatory drugs (NSAIDs)									
Celecoxib	Diarrhea	7	2.4	4.3	9.3	−28.6	0.4	3.3 (3.7, 14.9)	2.0
Celecoxib	Vomiting	7	1.9	3.1	9.3	−47.0	0.7	4.4 (5.1, 23.5)	2.8
Celecoxib	Constipation	7	0.8	1.4	9.3	−76.1	1.6	10.0 (11.2, 71.2)	5.5
Ibuprofen	Renal failure	13	1.9	1.7	8.3	−55.5	0.8	9.4 (4.6, 126.0)	3.3
Ibuprofen	Hypotension	8	1.7	1.2	5.1	−41.4	0.5	4.3 (3.1, 24.4)	2.3
Ibuprofen	Pneumonia	16	4.8	1.5	10.2	−38.0	0.3	5.5 (2.3, 80.5)	2.2
VRZ + Immunosuppressants									
Azathioprine	Pneumonia	27	4.8	3.7	9.3	−9.9	0.2	6.1 (2.7, 167.1)	2.1
Azathioprine	Diarrhea	25	2.3	5.5	8.7	−9.8	0.2	2.9 (2.9, 47.5)	2.2
Azathioprine	Renal failure	23	1.9	1.0	8.0	−63.6	1.5	10.8 (5.6, 287.0)	7.3
Azathioprine	Anemia	25	1.8	2.0	8.7	−57.1	1.4	9.0 (6.7, 251.6)	6.9
Azathioprine	Erythema	11	1.0	1.3	3.8	−39.5	0.7	3.7 (5.3, 29.0)	3.1
Dexamethasone	Condition aggravated	123	3.3	0.9	8.2	−48.2	0.4	4.1 (2.6, 376.8)	5.0
Dexamethasone	Hypokalemia	70	1.0	0.7	4.7	−62.8	0.7	13.4 (4.9, 1,000.5)	6.1
Hydrocortisone	Anemia	67	1.6	1.9	6.8	−47.8	0.8	5.2 (4.3, 313.7)	6.7
Methotrexate	Febrile neutropenia	104	2.8	1.3	9.1	−54.8	1.0	30.6 (4.4, 4441.6)	9.7
Methotrexate	Sepsis	51	2.5	1.4	4.5	−11.2	0.2	8.1 (2.4, 473.2)	2.5
Methotrexate	Neutropenia	54	2.2	1.2	4.7	−27.3	0.6	7.9 (2.9, 485.1)	4.6
Methotrexate	Anemia	41	1.8	1.3	3.6	−14.4	0.7	3.8 (2.8, 125.0)	4.6
Methotrexate	Pancytopenia	50	1.6	1.2	4.4	−36.0	0.5	16.5 (3.7, 1,076.1)	4.2
Methotrexate	Abdominal pain	32	0.9	1.7	2.8	−7.2	0.3	2.5 (3.0, 42.5)	2.7
Tacrolimus	Graft versus host disease	141	0.7	1.3	4.6	−57.0	0.4	81.7 (2.6, 15,031.4)	5.3
Tocilizumab	Off-label use	37	6.3	15.0	33.3	−36.2	0.4	8.7 (5.2, 442.1)	3.4
VRZ + other antibacterial drugs									
Amphotericin B	Product use issue	88	1.0	1.0	2.5	−17.9	0.2	2.1 (4.1, 60.9)	2.9
Ceftazidime	Condition aggravated	73	3.4	2.8	13.7	−54.5	0.8	7.6 (5.2, 613.1)	6.4
Ceftazidime	Product use issue	26	1.1	0.9	4.9	−58.8	1.0	4.0 (5.7, 82.0)	5.3
Ceftriaxone	Cardiac arrest	21	0.7	1.7	4.6	−47.6	0.5	8.3 (3.6, 175.1)	3.1
Cefuroxime	Condition aggravated	12	3.6	2.3	17.4	−65.8	1.7	18.4 (9.2, 267.6)	6.5
Ciprofloxacin	Condition aggravated	121	3.3	2.4	8.9	−35.9	0.3	4.7 (2.8, 478.5)	4.4
Ciprofloxacin	Anemia	68	1.6	2.0	5.0	−27.8	0.2	3.5 (3.0, 161.8)	2.9
Ciprofloxacin	Constipation	47	0.7	1.7	3.5	−33.1	0.1	2.3 (2.5, 47.8)	2.3
Clarithromycin	Hypotension	17	1.7	1.5	6.2	−48.4	0.9	5.1 (4.7, 75.5)	4.2
Levofloxacin	Febrile neutropenia	95	2.9	2.0	6.4	−24.7	0.0	18.3 (3.1, 2033.9)	2.2
Linezolid	Condition aggravated	104	3.3	2.0	11.2	−52.6	0.4	4.6 (3.1, 382.2)	4.8

Abbreviation: AE, adverse event; CRR, combination risk ratio; DDIs, drug–drug interactions; DE, reported number of adverse events when the drugs are used in combination; N, number; PRR, proportional reporting ratio; PT, preferred term; VRZ, voriconazole.

^a^ Criterion for positive safety signals related to DDIs of the four frequency statistical models: R_diff < 0; Ω_0.25_ > 0; CRR > 0, PRR >2, χ2 > 4; χ > 2.

^b^ Reported number of target AEs when two drugs are used together, and its meaning equivalent to DE, shown in Additional File 1.

^c^ R_diff: the observed AE frequency of combined drug use (RE) was greater than the expected AE frequency (the sum of the occurrence frequency when each drug was used separately).

^d^ Ω_0.25_: the upper limit of bilateral 95% confidence interval of Ω.

### 3.3 Correlation analysis

**FIGURE 2:**
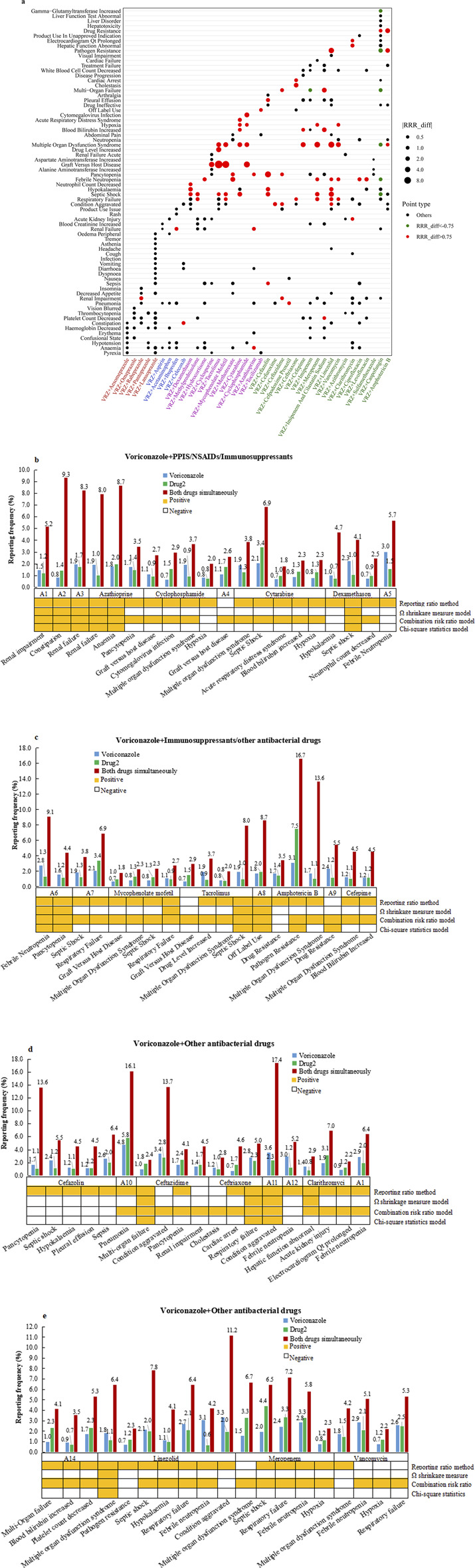
Correlation analysis results between drug(s) and target AEs with positive DDI signals. **(A)** Bubble chart depicting correlation analysis results of the 262 adverse events. Red spots represent RRR_diff values >0.75, indicating that the significant positive correlation between the target AE and the combined use of the two drugs was greater than that of the two single drugs. Green spots represent RRR_diff values < −0.75 and indicate a significant negative correlation between the target AE and the two drugs used alone when more than two drugs are used together. The larger the bubble, the greater the absolute value of the RRR_diff value, indicating a greater correlation between the drug(s) and the target AE. **(B‐E)**: Reporting rates of 77 adverse events positively correlated with DDIs when VRE in combination with target drugs was compared with when VRE alone was used. The orange square under the bar chart indicates a positive safety signal related to the DDI calculated by the corresponding model. (Abbreviations: A1: rabeprazole; A2: celecoxib; A3: ibuprofen; A4: cyclosporine; A5: hydrocortisone; A6: methotrexate; A7: methylprednisolone; A8: tocilizumab; A9: caspofungin; A10: cefpodoxime proxetil; A11: cefuroxime; A12: ciprofloxacin; A13: levofloxacin; A14: imipenem and cilastatin sodium).

The correlation analysis results between the target drug and the target AEs with positive DDI signals are shown in Figure 2A. Some 77 (29.4%) target AEs were significantly positively correlated with DDIs when VRZ was used in combination, leading to an increased risk of AEs (Table 3). The reporting rates and four frequency statistical model distributions of these 77 AEs are presented in Figures 2B–E. The reporting rate of all 77 AEs significantly positively correlated with DDIs increased when VRZ was used in combination with the target drug compared with when VRZ was used alone; close attention should thus be given to these target AEs. For VRZ in combination with tacrolimus, mycophenolate mofetil, or cyclophosphamide, graft-versus-host disease should be of particular concern because this AE had the strongest correlation with the drug interaction, and multiple organ dysfunction syndrome deserves great attention when VRZ is used in combination with meropenem, cefepime, or mycophenolate mofetil.

**TABLE 3:** Results of target AEs with positive correlation with DDIs when VRZ was used in combination with the target drug^a^ (n = 77).

Drug	Adverse event (PT)	AE reports(N)^b^	RRR_1_	RRR_2_	RRR_E_	Delta_RRR	RRR_diff^c^
VRZ + Proton pump inhibitors (PPIs)							
Rabeprazole	Renal impairment	328	3.7	2.9	13.1	9.8	1.2
VRZ + non-steroidal anti-inflammatory drugs (NSAIDs)							
Celecoxib	Constipation	181	0.8	1.4	9.4	8.3	1.0
Ibuprofen	Renal failure	434	2.6	2.4	11.2	8.7	1.1
VRZ + immunosuppressants							
Azathioprine	Renal failure	424	2.6	1.3	10.8	8.8	1.1
Azathioprine	Anemia	391	1.8	2.0	9.0	7.0	0.9
Azathioprine	Pancytopenia	384	6.4	5.4	12.9	7.0	0.9
Cyclophosphamide	Graft versus host disease	232	29.3	24.8	73.0	45.9	5.7
Cyclophosphamide	Cytomegalovirus infection	139	8.1	19.1	36.6	22.9	2.8
Cyclophosphamide	Multiple organ dysfunction syndrome	402	17.3	8.3	33.5	20.7	2.6
Cyclophosphamide	Hypoxia	175	4.9	4.5	12.0	7.3	0.9
Cyclosporine	Graft versus host disease	227	29.0	45.7	69.7	32.4	4.0
Cytarabine	Multiple organ dysfunction syndrome	397	17.2	11.5	35.1	20.7	2.6
Cytarabine	Septic shock	435	10.2	16.7	33.9	20.4	2.5
Cytarabine	Acute respiratory distress syndrome	143	7.7	11.0	20.3	10.9	1.4
Cytarabine	Blood bilirubin increased	179	5.9	9.1	15.8	8.3	1.0
Cytarabine	Hypoxia	169	4.8	7.6	13.9	7.7	1.0
Dexamethasone	Hypokalemia	209	4.5	3.3	20.9	17.0	2.1
Dexamethasone	Septic shock	474	11.1	5.1	20.0	11.9	1.5
Dexamethasone	Neutrophil count decreased	156	4.2	5.3	13.8	9.1	1.1
Hydrocortisone	Febrile neutropenia	645	9.9	5.1	18.8	11.3	1.4
Methotrexate	Febrile neutropenia	597	9.2	4.4	30.2	23.4	2.9
Methotrexate	Pancytopenia	344	6.0	4.5	16.4	11.1	1.4
Methylprednisolone	Septic shock	461	10.9	6.4	21.6	13.0	1.6
Methylprednisolone	Respiratory failure	545	7.0	5.2	16.2	10.0	1.2
Mycophenolate Mofetil	Graft versus host disease	196	25.4	32.5	101.9	72.9	9.0
Mycophenolate Mofetil	Multiple organ dysfunction syndrome	372	16.5	11.9	38.1	23.9	3.0
Mycophenolate Mofetil	Septic shock	453	10.8	7.8	20.9	11.5	1.4
Mycophenolate Mofetil	Respiratory failure	544	7.1	5.0	14.2	8.2	1.0
Tacrolimus	Graft versus host disease	129	17.7	34.8	122.0	95.8	11.9
Tacrolimus	Drug level increased	365	23.3	14.4	46.8	27.9	3.5
Tacrolimus	Multiple organ dysfunction syndrome	335	15.7	10.6	34.9	21.8	2.7
Tacrolimus	Septic shock	425	10.8	7.3	17.6	8.6	1.1
Tocilizumab	Off-label use	1,406	1.9	4.6	10.3	7.0	0.9
VRZ + other antibacterial drugs							
Amphotericin B	Drug resistance	268	12.0	8.7	32.5	22.2	2.7
Amphotericin B	Pathogen resistance	126	16.5	14.9	33.6	17.9	2.2
Amphotericin B	Multiple organ dysfunction syndrome	348	16.7	24.0	27.0	6.6	0.8
Caspofungin	Drug resistance	360	14.7	16.1	22.0	6.6	0.8
Cefazolin	Pancytopenia	370	6.3	3.9	50.7	45.5	5.6
Cefazolin	Septic shock	518	11.7	5.9	26.8	18.0	2.2
Cefazolin	Hypokalemia	271	5.6	4.7	20.3	15.1	1.9
Cefazolin	Pleural effusion	257	3.8	3.8	14.5	10.7	1.3
Cefazolin	Sepsis	574	4.7	3.6	11.4	7.2	0.9
Cefepime	Multiple organ dysfunction syndrome	404	17.6	15.0	41.1	24.8	3.1
Cefepime	Blood bilirubin increased	192	6.3	5.2	13.5	7.7	1.0
Cefpodoxime Proxetil	Pneumonia	1,081	3.1	3.8	10.5	7.1	0.9
Ceftazidime	Multi-organ failure	217	8.7	15.6	20.9	8.7	1.1
Ceftazidime	Condition aggravated	732	2.7	2.2	10.8	8.4	1.0
Ceftazidime	Pancytopenia	363	6.3	9.0	15.3	7.6	0.9
Ceftazidime	Renal impairment	307	3.6	4.3	11.4	7.4	0.9
Ceftriaxone	Cholestasis	270	13.3	10.5	30.7	18.8	2.3
Ceftriaxone	Cardiac arrest	161	1.7	3.8	10.5	7.8	1.0
Ceftriaxone	Respiratory failure	625	7.6	6.0	13.4	6.6	0.8
Cefuroxime	Condition aggravated	812	2.8	1.8	13.6	11.3	1.4
Ciprofloxacin	Febrile neutropenia	630	9.9	4.1	17.4	10.4	1.3
Clarithromycin	Hepatic function abnormal	321	8.4	4.9	17.0	10.4	1.3
Clarithromycin	Acute kidney injury	434	2.8	4.5	10.0	6.4	0.8
Clarithromycin	Electrocardiogram Qt prolonged	203	5.2	7.1	12.6	6.5	0.8
Imipenem and cilastatin sodium	Multi-organ failure	223	8.7	20.2	36.1	21.7	2.7
Imipenem and cilastatin sodium	Blood bilirubin increased	206	6.4	4.8	24.7	19.0	2.4
Imipenem and cilastatin sodium	Platelet count decreased	389	3.4	4.5	10.3	6.4	0.8
Levofloxacin	Febrile neutropenia	606	9.5	6.5	21.3	13.3	1.6
Linezolid	Multiple organ dysfunction syndrome	393	16.6	10.5	58.8	45.3	5.6
Linezolid	Pathogen resistance	153	17.6	29.6	56.1	32.5	4.0
Linezolid	Septic shock	462	10.5	9.8	38.7	28.5	3.5
Linezolid	Hypokalemia	241	5.0	4.3	18.3	13.7	1.7
Linezolid	Respiratory failure	587	7.3	5.6	17.2	10.8	1.3
Linezolid	Febrile neutropenia	662	10.2	2.1	13.9	7.8	1.0
Linezolid	Condition aggravated	720	2.6	1.5	8.7	6.7	0.8
Meropenem	Multiple organ dysfunction syndrome	317	14.1	29.8	60.8	38.9	4.8
Meropenem	Septic shock	403	9.7	21.7	31.9	16.2	2.0
Meropenem	Respiratory failure	501	6.5	8.9	19.1	11.4	1.4
Meropenem	Febrile neutropenia	583	9.4	11.0	19.2	9.0	1.1
Meropenem	Hypoxia	157	4.5	6.9	13.4	7.7	1.0
Vancomycin	Multiple organ dysfunction syndrome	355	16.0	13.5	38.4	23.6	2.9
Vancomycin	Febrile neutropenia	582	9.5	7.1	17.0	8.6	1.1
Vancomycin	Hypoxia	151	4.4	7.0	13.3	7.5	0.9
Vancomycin	Respiratory failure	523	6.9	6.6	14.2	7.5	0.9

Abbreviation: DDIs, drug–drug interactions; DE, reported number of adverse events when drugs are used in combination; PT, preferred term; RRR, proportional reporting ratio; RRR_1_, proportional reporting ratio for VRZ; RRR_2_, proportional reporting ratio for the target drug; RRR_E_, proportional reporting ratio for adverse events when drugs are used in combination; Delta_RRR, difference between the RRRE value when two drugs are used together and mean RRR_1_ and RRR_2_ when two drugs are used alone; VRZ, voriconazole.

^a^ Only data positively correlated with the drug interaction of VRZ and target drug are demonstrated in Table 3.

^b^ Reported number of target AEs when two drugs are used together, and its meaning equivalent to DE, are shown in Additional File 1.

^c^ RRR_diff value > 0.75 indicates that the target AE is positively correlated with DDIs when VRZ was used in combination. The higher the RRR_diff value, the greater the correlation between the drug(s) and the target AE.

On the other hand, according to the statistical analysis results of this study, we did not find a significant correlation with target AEs related to DDIs between VRZ and aesomeprazole, lansoprazole, omeprazole, pantoprazole, acetaminophen, aspirin, azithromycin, imipenem, or sulfamethoxazole. Seven (2.7%) target AEs were negatively correlated with DDIs when VRZ was used in combination, so the risk of the target AEs might thus be greater when the two drugs are used alone than when VRZ is used in combination.

### 3.4 Influence of sex and age on the AEs related to DDIs

**FIGURE 3:**
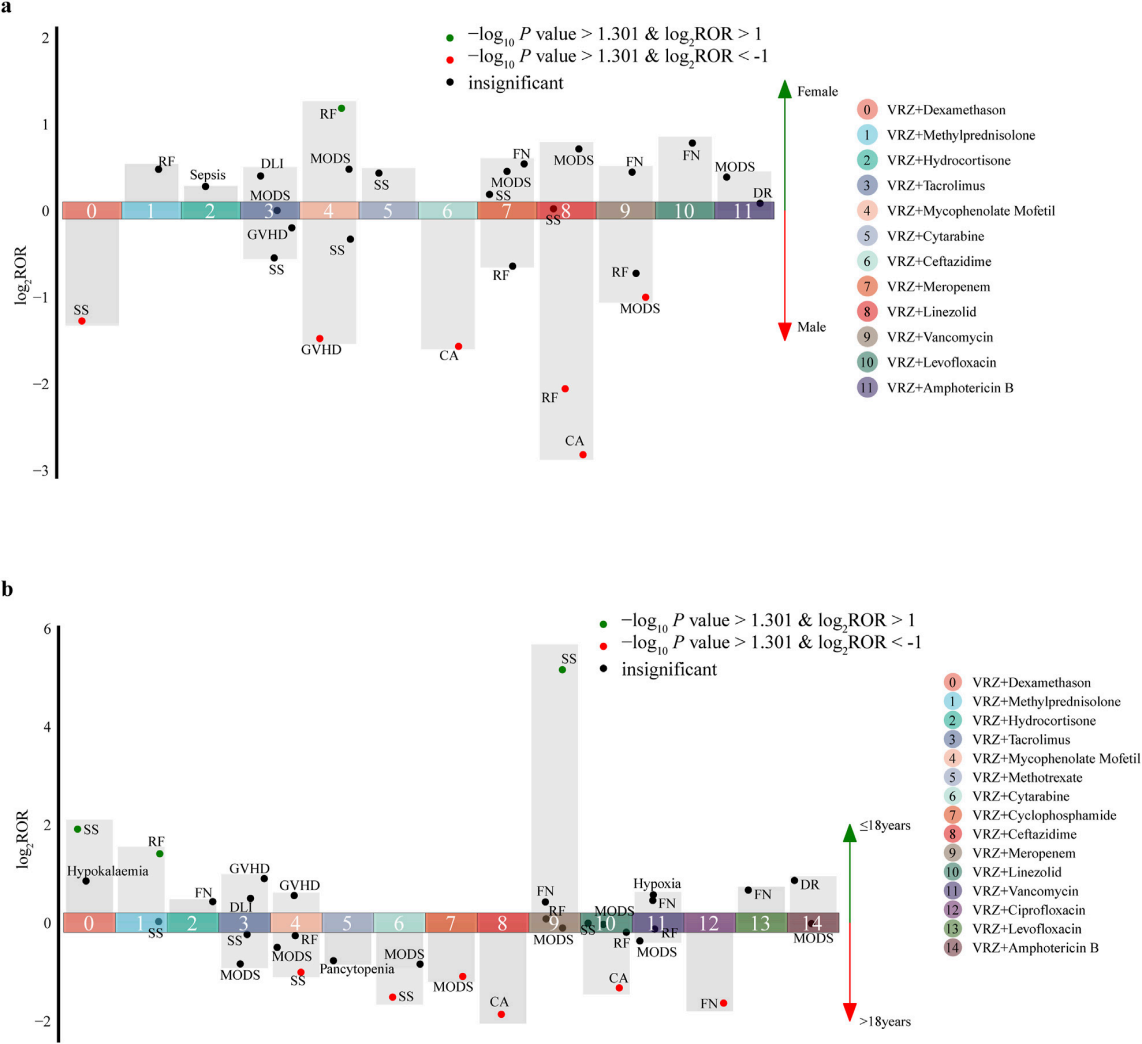
Influence of sex and age on the incidence of AEs related to drug‒drug interactions (DDIs). **(A)** sex; **(B)** age. Each spot represents a specific AE related to DDIs. Green spots represent AEs more frequently associated with female (or ≤18 years) patients; red spots represent AEs more frequently associated with male (or >18 years) patients. (Abbreviations: CA, condition aggravated; DLI, drug level increased; DR, drug resistance; FN, febrile neutropenia; GVHD, graft-versus-host disease; MODS, multiple organ dysfunction syndrome; RF, respiratory failure; SS, septic shock).

The 77 AEs positively correlated with DDIs were included to evaluate the influence of sex and age; 27 target AEs in the sex group and 34 in the age group met the inclusion criteria for statistical analysis (Tables 4 and 5, Figures 3A and B). Five drugs had significant sex differences in target AEs related to DDIs. Male patients might be more susceptible than female patients to these target AEs (6/27 vs. 1/27), and close attention should be given to disease aggravation and respiratory failure when VRZ is used in combination with linezolid. For female patients, respiratory failure should be considered first when VRZ is used in combination with mycophenolate mofetil.

**TABLE 4:** Comparison of the risk of AEs related to DDIs between female and male patients.

Drug	Adverse event (PT)	Female		Male		χ	*p*	ROR (95%CI)	Log_10_ *P*	Log_2_ROR
		Target AE	Other AEs	Target AE	Other AEs					
VRZ + immunosuppressants										
Cytarabine	Septic shock	43	869	52	1,417	2.03	0.154	1.35 (0.89–2.04)	0.812	0.431
Dexamethasone	Septic shock	16	1,019	41	1,078	9.36	0.002	0.41 (0.23–0.74)	2.699	−1.276
Hydrocortisone	Sepsis	23	526	29	804	0.46	0.498	1.21 (0.69–2.12)	0.303	0.278
Methylprednisolone	Respiratory failure	40	712	58	1,436	2.48	0.116	1.39 (0.92–2.10)	0.936	0.476
Mycophenolate mofetil	Respiratory failure	53	831	46	1,636	16.61	<0.000	2.27 (1.51–3.40)	3.000	1.182
Mycophenolate mofetil	Septic shock	21	863	50	1,632	0.77	0.381	0.79 (0.47–1.33)	0.419	−0.332
Mycophenolate mofetil	Multiple organ dysfunction syndrome	29	855	40	1,642	1.80	0.179	1.39 (0.86–2.26)	0.747	0.478
Mycophenolate mofetil	Graft versus host disease	10	874	52	1,630	9.44	0.002	0.36 (0.18–0.71)	2.699	−1.479
Tacrolimus	Septic shock	27	1,510	72	2,753	2.82	0.093	0.68 (0.44–1.07)	1.032	−0.549
Tacrolimus	Drug level increased	47	1,490	66	2,759	2.05	0.152	1.32 (0.90–1.93)	0.818	0.399
Tacrolimus	Multiple organ dysfunction syndrome	37	1,500	68	2,757	0.00	1.000	1.00 (0.67–1.50)	0.000	0.000
Tacrolimus	Graft versus host disease	39	1,498	82	2,743	0.49	0.483	0.87 (0.59–1.28)	0.316	−0.199
VRZ + other antibacterial drugs										
Amphotericin B	Multiple organ dysfunction syndrome	38	1,626	55	3,075	1.58	0.208	1.31 (0.86–1.98)	0.682	0.386
Amphotericin B	Drug resistance	45	1,619	80	3,050	0.09	0.759	1.06 (0.73–1.53)	0.120	0.084
Ceftazidime	Condition aggravated	6	149	67	560	6.82	0.009	0.34 (0.14–0.79)	2.046	−1.571
Levofloxacin	Febrile neutropenia	47	569	42	873	6.21	0.013	1.72 (1.12–2.64)	1.886	0.780
Linezolid	Condition aggravated	13	872	88	835	55.72	<0.000	0.14 (0.08–0.26)	3.000	−2.822
Linezolid	Respiratory failure	11	874	46	877	20.71	<0.000	0.24 (0.12–0.47)	3.000	−2.059
Linezolid	Septic shock	34	851	35	888	0.00	0.956	1.01 (0.63–1.64)	0.020	0.020
Linezolid	Multiple organ dysfunction syndrome	34	851	22	901	3.20	0.074	1.64 (0.95–2.82)	1.131	0.710
Meropenem	Febrile neutropenia	52	1,105	62	1914	3.83	0.050	1.45 (1.00–2.12)	1.301	0.539
Meropenem	Respiratory failure	37	1,120	97	1879	5.22	0.022	0.64 (0.44–0.94)	1.658	−0.644
Meropenem	Septic shock	49	1,108	74	1902	0.47	0.495	1.14 (0.79–1.64)	0.305	0.185
Meropenem	Multiple organ dysfunction syndrome	53	1,104	67	1909	2.81	0.094	1.37 (0.95–1.98)	1.027	0.452
Vancomycin	Febrile neutropenia	47	1,371	60	2,379	2.43	0.119	1.36 (0.92–2.00)	0.924	0.443
Vancomycin	Respiratory failure	31	1,387	87	2,352	5.77	0.016	0.60 (0.40–0.92)	1.796	−0.727
Vancomycin	Multiple organ dysfunction syndrome	20	1,398	68	2,371	7.63	0.006	0.50 (0.30–0.82)	2.222	−1.003

Abbreviation: AE, adverse event; CI: confidence interval; DDIs: drug-drug interactions; PT: preferred term; ROR: reporting odds ratio; VRZ: voriconazole.

Statistically significant results highlighted in bold: log_2_ROR > 1 or log_2_ROR < −1, and Pearson’s chi-squared test with *P* < 0.05. ROR >1 indicates female patients are more likely to have a higher risk of target AEs, and 0 < ROR < 1 indicates male patients tends to have a higher risk of target AEs.

**TABLE 5:** Comparison of risk of AEs related to DDIs between ≤18 years and >18 years patients.

Drug	Adverse event (PT)	≤18 years patients		>18 years patients		χ	*p*	ROR (95%CI)	Log_10_ *P*	Log_2_ROR
		Target AE	Other AEs	Target AE	Other AEs					
VRZ + immunosuppressants										
Cyclophosphamide	Multiple organ dysfunction syndrome	13	813	38	1,112	5.7	**0.017**	0.47 (0.25–0.88)	**1.770**	**−1.096**
Cytarabine	Septic shock	19	987	81	1,469	18.1	<0.000	0.35 (0.21–0.58)	**3.000**	**−1.518**
Cytarabine	Multiple organ dysfunction syndrome	15	991	41	1,509	3.8	**0.052**	0.56 (0.31–1.01)	1.284	−0.844
Dexamethasone	Septic shock	61	636	41	1,603	46.0	**<0.000**	3.75 (2.50–5.63)	**3.000**	**1.907**
Dexamethasone	Hypokalaemia	30	667	40	1,604	5.9	0.015	1.80 (1.11–2.92)	1.824	0.851
Hydrocortisone	Febrile neutropenia	26	506	30	786	1.2	0.276	1.35 (0.79–2.30)	0.559	0.429
Methotrexate	Pancytopenia	23	641	27	440	3.5	0.062	0.58 (0.33–1.03)	1.208	−0.774
Methylprednisolone	Respiratory failure	47	529	55	1,639	24.2	**<0.000**	2.65 (1.77–3.96)	**3.000**	**1.405**
Methylprednisolone	Septic shock	19	557	55	1,639	0.0	0.952	1.02 (0.60–1.73)	0.021	0.024
Mycophenolate mofetil	Respiratory failure	25	728	80	1938	0.6	0.429	0.83 (0.53–1.31)	0.368	−0.266
Mycophenolate mofetil	Septic shock	13	740	69	1949	5.5	**0.019**	0.50 (0.27–0.90)	**1.721**	**−1.011**
Mycophenolate mofetil	Multiple organ dysfunction syndrome	17	736	64	1954	1.6	0.204	0.71 (0.41–1.21)	0.690	−0.504
Mycophenolate mofetil	Graft versus host disease	26	727	48	1970	2.4	0.119	1.47 (0.90–2.38)	0.924	0.554
Tacrolimus	Septic shock	27	1,280	83	3,330	0.6	0.456	0.85 (0.55–1.31)	0.341	−0.241
Tacrolimus	Drug level increased	126	1,181	240	3,173	9.0	0.003	1.41 (1.13–1.77)	2.523	0.496
Tacrolimus	Multiple organ dysfunction syndrome	21	1,286	97	3,316	5.9	0.015	0.56 (0.35–0.90)	1.824	−0.841
Tacrolimus	Graft versus host disease	58	1,249	83	3,330	13.1	<0.000	1.86 (1.32–2.62)	3.000	0.898
VRZ + other antibacterial drugs										
Amphotericin B	Multiple organ dysfunction syndrome	36	1714	69	3,243	0.0	0.950	0.99 (0.66–1.48)	0.022	−0.019
Amphotericin B	Drug resistance	66	1,684	70	3,242	12.0	0.001	1.82 (1.29–2.55)	3.000	0.860
Ceftazidime	Condition aggravated	5	150	68	559	5.7	**0.017**	0.27 (0.11–0.69)	**1.770**	**−1.868**
Ciprofloxacin	Febrile neutropenia	22	1994	49	1,425	21.3	**<0.000**	0.32 (0.19–0.53)	**3.000**	**−1.640**
Levofloxacin	Febrile neutropenia	42	544	53	1,089	4.8	0.029	1.59 (1.04–2.41)	1.538	0.666
Linezolid	Condition aggravated	8	289	96	1,380	6.5	**0.011**	0.40 (0.19–0.83)	**1.959**	**−1.329**
Linezolid	Respiratory failure	9	288	51	1,425	0.1	0.712	0.87 (0.43–1.79)	0.148	−0.196
Linezolid	Septic shock	12	285	61	1,415	0.0	0.942	0.98 (0.52–1.84)	0.026	−0.034
Linezolid	Multiple organ dysfunction syndrome	10	287	50	1,426	0.0	0.986	0.99 (0.50–1.98)	0.006	−0.009
Meropenem	Febrile neutropenia	38	816	80	2,303	2.3	0.144	1.34 (0.90–1.99)	0.842	0.423
Meropenem	Respiratory failure	40	814	106	2,277	0.1	0.776	1.06 (0.73–1.53)	0.110	0.078
Meropenem	Septic shock	121	733	11	2,372	302.0	**<0.000**	35.60 (19.10–66.35)	**3.000**	**5.154**
Meropenem	Multiple organ dysfunction syndrome	34	820	102	2,281	0.1	0.709	0.93 (0.62–1.38)	0.149	−0.109
Vancomycin	Febrile neutropenia	46	1,208	73	2,627	2.7	0.099	1.37 (0.94–1.99)	1.004	0.455
Vancomycin	Respiratory failure	37	1,217	87	2,613	0.2	0.648	0.91 (0.62–1.35)	0.188	−0.131
Vancomycin	Multiple organ dysfunction syndrome	26	1,228	72	2,628	1.2	0.264	0.77 (0.49–1.22)	0.578	−0.372
Vancomycin	Hypoxia	24	1,394	28	2,411	5.1	0.024	1.48 (0.86–2.57)	1.620	0.568

Abbreviation: AE, adverse event; CI, confidence interval, DDIs: drug–drug interactions; PT, preferred term; ROR, reporting odds ratio; VRZ, voriconazole.

Statistically significant results highlighted in bold: log_2_ROR > 1 or log_2_ROR < −1, and Pearson’s chi-squared test with *P* < 0.05. ROR > 1 indicates ≤18 years is more likely to have a higher risk of target AEs, and 0 < ROR < 1 indicates >18 years tend to have a higher risk of target AEs.

For age groups, nine drugs had significant age differences in target AEs related to DDIs. Compared with ≤18-year-old patients, >18-year-old patients tended to have detected AEs (6/34 vs. 3/34), and more attention should be given to disease aggravation, febrile neutropenia and septic shock when VRZ is used in combination with ceftazidime, ciprofloxacin, and cytarabine, respectively. For ≤18-year-old patients, septic shock should be considered when VRZ is used in combination with meropenem and dexamethasone. Moreover, on the basis of the statistical analysis results of this study, we did not find a significant difference in sex and age for the AEs related to DDIs associated with the PPIs and NSAIDs included in this study.

## 4 Discussion

To our knowledge, this is the first pharmacovigilance study to comprehensively assess the DDIs between VRZ and 38 drugs commonly prescribed in clinical practice. Signal mining via FAERS could provide a reference for safety monitoring when VRZ is combined with other drugs in clinical practice and could serve as a starting point for further analysis. Owing to the voluntary nature of FAERS reporting, underreporting, overreporting, or missing information was unavoidable (Meng et al., 2022; Allegra et al., 2020), and due to the technical limitations and the constant changes of the OpenVigil FDA API, there might also be some imbalance in the data extracted from the database. During our analysis, we found that the imbalance of the data was approximately 7%. For a specific positive signal result, the more models that detected it, the more reliable the result; therefore, to ensure the consistency and reliability of the drug interaction results, four models were used to analyse the data simultaneously. The reporting ratio method and combined risk ratio model were used to compare the related parameters when drugs were used in combination and alone and then to evaluate the influence of DDI (Norén et al., 2008). The Ω shrinkage measure model and chi-square statistics model were used to compare the observation values and the expected values of the related parameters. The false-positive rate and sensitivity of random fluctuations for DDI results could be effectively controlled with the use of a chi-square statistics model and an Ω shrinkage measure model, respectively (Gosho et al., 2017). Overall, real-world research from a database is more feasible than drug clinical trials for drug interaction studies because clinical trials needs to consider stricter entry criteria, more limited patients, and greater risk. In this study, potential AEs related to DDIs of VRZ were detected, which can provide a reference for safety monitoring when VRZ is used in combination with other drugs in clinical practice.

Voriconazole in various drug combinations has the potential for multiple adverse drug–drug interactions; describing drug interactions in detail in the literature is often difficult because of large differences in how reactions are defined and the severity of reactions between individuals. In addition, although VRZ interactions between drugs are theoretically recognizable, all of these interactions may not necessarily be clinically significant (Glotzbecker et al., 2012). Therefore, this study focused on the AEs that had the strongest correlation with the drug interaction and its influence.

Age and sex may influence VRZ levels and play crucial roles in the occurrence of AEs (Yu et al., 2016). One study focused on sex differences in adverse drug events using FAERS and detected sex differences in AEs (i.e., alopecia, amnesia, and urticaria) (Yu et al., 2016). Our analysis revealed that sex and age might influence the occurrence of some adverse reactions, especially when VRZ is combined with some immunosuppressants or other antibacterial drugs. For example, when VRZ is combined with mycophenolate mofetil, the risk of respiratory failure might be greater in female patients, but the risk of graft-versus-host disease might be greater in male patients, and the risk of septic shock might be greater in >18-year-old patients. Sex and age could influence VRZ trough concentrations, and the sex effect on drug concentrations may be due to sex differences in CYP-mediated metabolism, the influence of sex hormones on drug absorption, and differences in fat percentage with respect to body composition (Allegra et al., 2020); sex differences could also be the result of different doses/kg of body weight. Thus, the recommended weight-based VRZ dosing should also consider a patient’s sex to avoid underexposure, especially in women. In addition, when oral administration is not prescribed by body weight, BMI plays a predictive role, and a lower BMI value predicts higher drug concentrations (Shao et al., 2017).

In terms of age, one study on healthy volunteers revealed that the maximum VRZ concentration and area under the curve were greater in elderly male subjects and in women than in younger men, as increasing age was a predictive factor of a higher VRZ trough via the intravenous route. Decreased metabolic clearance in elderly individuals could strongly influence the drug concentration, especially given that VRZ is a renally excreted drug; therefore, drug-impaired renal function should be judged in clinical practice by examining creatinine levels (Allegra et al., 2020).

In this study, we found that VRZ in combination with cyclosporine, tacrolimus, mycophenolate mofetil, and cyclophosphamide could lead to a significant increase in the probability of graft-versus-host disease, with a strong correlation with DDIs; these results are consistent with those of Groll et al. (2017). The modulation of cytochrome P450 3A4 and drug transporters such as P-gp may alter the blood levels of both antimicrobial agents and immunosuppressants, and the use of antimicrobial agents can interfere with the metabolism of immunosuppressants, which may put patients at risk of developing severe AEs due to unwanted increases or decreases in the serum levels of immunosuppressive agents (Shao et al., 2017; Bhagat et al., 2021). Therefore, interactions between immunosuppressants and antimicrobial agents can cause non-infectious complications like graft-vs.-host-disease (GVHD) flares (Champion et al., 2006). The appropriate dosing and delivery of antimicrobial agents in immunosuppressed patients with organ dysfunction is a major therapeutic challenge. It is desirable, from a clinical perspective, to avoid unnecessarily high exposure to immunosuppressants (Thomas et al., 2009; Dong et al., 2024). A previous study reported that VRZ treatment led to a dramatic increase in tacrolimus concentration, which required discontinuation despite the manufacturer’s guidelines recommending a one-third reduction in tacrolimus dosage. Therefore, the drug dose needs to be adjusted on the basis of the results of therapeutic drug monitoring to ensure that patients avoid serious AEs when VRZ is utilized in combination with immunosuppressants (Beata et al., 2017).

A previous study demonstrated that many antibiotics, including imipenem, cefepime, ceftazidime, vancomycin, and levofloxacin, are unlikely to cause DDIs because they are primarily eliminated in their unchanged form via glomerular filtration (Job et al., 2016). However, we found that the occurrence of adverse reactions was strongly related to DDIs when VRZ was used in combination with other antibiotics. For example, the frequency of multiple organ dysfunction syndrome might be approximately four times greater when VRZ is combined with linezolid or meropenem and is strongly correlated with DDIs. When linezolid combined with VRZ treatment increases VRZ clearance to between 250% and 700% and serum antifungal concentrations decrease clinically, the effectiveness of antifungal therapy is lost in 80% of cases (Idoate et al., 2019). Therefore, the combination of linezolid and voriconazole is not recommended if no other clinical alternative exists, and VRZ pharmacokinetic monitoring is recommended to ensure the effectiveness of antifungal treatment.

A combination of VRZ and glucocorticoids can prevent invasive fungal infections, but the concomitant administration of glucocorticoids and VRZ might be challenging due to the high propensity for DDIs (Gergis et al., 2010). In this study, septic shock and respiratory failure should receive close attention when VRZ is combined with methylprednisolone. The reason could be that VRZ markedly increases the plasma concentrations of dexamethasone and methylprednisolone, leading to AEs when dexamethasone or methylprednisolone is used in combination with VRZ. Therefore, the dose of dexamethasone or methylprednisolone should be reduced to maintain approximately similar exposures, and close attention should be given to the symptoms of patients when glucocorticoids and voriconazole are used in combination in the clinic (Li M. et al., 2018).

Both VRZ and PPIs are metabolized primarily by CYP2C19, and the VRZ concentration increases with the administration of PPIs (Tian et al., 2021), possibly increasing the risk of adverse drug reactions. To our knowledge, there are few reports regarding the interaction of VRZ with PPIs, except for omeprazole, such as lansoprazole, pantoprazole, and rabeprazole (Tian et al., 2021). Our study found that VRZ in combination with rabeprazole could lead to a significant increase in the probability of renal impairment, with a strong correlation with DDIs. VRZ-induced renal impairment has been reported by Turner et al. (2015). Although we did not find a significant correlation with target AEs related to DDIs between VRZ and omeprazole, all four models indicated that a confused state, thrombocytopenia, blurred vision, and decreased haemoglobin were positive safety indicators, and the reporting rates of those AEs were increased by three, two, two, and two times in concurrent use with VRZ, respectively. Dosage adjustments are recommended to prevent drug interactions from occurring when VRZ is used in combination with PPIs (Beata et al., 2017).

Our analysis found that the reporting rates of constipation and renal failure increased significantly when VRZ was used in combination with celecoxib and ibuprofen, respectively, and that both rates were strongly correlated with DDI. These results might be explained by increased exposure to ibuprofen or celecoxib because the inhibition of CYP2C9 by VRZ may lead to an increased risk of impaired renal function or constipation, respectively (Li N. et al., 2018). The AEs and toxicity associated with NSAIDs should be closely monitored when taken in combination with VRZ, and a reduced dose of ibuprofen or celecoxib should be considered to reduce the risk of DDIs when used in combination with VRZ, especially when the initial dose is high (Li N. et al., 2018; Chen et al., 2022).

In this study, although 29.4% of the target AEs were positively correlated with DDIs when VRZ was used in combination, the target AEs (i.e., septic shock, multiple organ dysfunction syndrome, multiorgan failure, increased amma-glutamyltransferase activity, and pathogen resistance) were negatively correlated with DDIs when VRZ was combined with caspofungin. A previous retrospective study of signal detection for adverse drug reactions based on databases in Japan and the United States also revealed that the proportional reporting ratios of neutropenia, haemorrhagic cystitis, and alopecia tended to be reduced when VRZ was combined with cyclophosphamide (Zhao et al., 2021), but the strength of the correlation between adverse drug effects and DDIs was unclear. Different patients have different physiological and pathological statuses, and the related mechanism deserves further study, which may be related to the large inter- and individual variability in VRZ metabolism (Kim et al., 2010).

## 5 Limitations

This study had several limitations. First, the data presented in the OpenVigil FDA platform were incomplete and lacked detailed patient information, especially for patients with complications and concomitant medications; this limited our ability to further assess the patient’s disease status and the severity of the AEs. Second, this study evaluated only the interaction between two drugs; multidrug interactions should also be studied in the future, and further extraction of the original reports is necessary to obtain more information. Additionally, the mechanism of DDIs could not be assessed in this study because of factors such as a lack of information on drug doses and laboratory values; therefore, further studies are needed to confirm the AEs detected in this study.

## 6 Conclusion

Voriconazole, an inhibitor of CYP3A4, can interact with many drugs, which may result in changes in the activity of the drug and cause serious AEs. An understanding of VRZ drug‒drug interactions (DDIs) and therapeutic drug monitoring is important for providing effective antifungal therapy. Health professionals should be more cautious when VRZ is used concomitantly with other drugs due to the risk of DDIs. More important AEs related to DDIs should receive more attention when VRZ is used in combination with PPIs (renal impairment), NSAIDs (constipation and renal failure), immunosuppressants (graft versus host disease, septic shock) and other antibacterial drugs (multiple organ dysfunction syndrome, febrile neutropenia, and respiratory failure). The influence of sex and age differences in VRZ DDIs also needs to be considered during the risk assessment of AEs in clinical therapy.

## Data Availability

The raw data supporting the conclusions of this article will be made available by the authors, without undue reservation.
