# Supplementary material for: Clinical drug interactions between voriconazole and 38 other drugs: a retrospective analysis of adverse events
Source: Front Pharmacol. 2024 Sep 30;15:1292163. doi: 10.3389/fphar.2024.1292163 (PMC11471622; doi:10.3389/fphar.2024.1292163)
Supplement: Supplementary file 1 [file DataSheet1.docx]

**Supplemental material**

| **Supplemental Table 1: Parameters for the frequency statistical models** | | | |  |
| --- | --- | --- | --- | --- |
| Drugs | Count_target AE | Count_all other AEs | Total | |
| VRZ+ concomitant drug | D_E_ | d_E_ | D_b_ | |
| Only VRZ | D_1_ | d_1_ | D_L_ | |
| Only the concomitant drug | D_2_ | d_2_ | D_e_ | |
| Neither VRZ nor the concomitant drug | D_N_ | d_N_ | d_e_ | |
| Total | E | e | n (All Reports) | |
| Abbreviation: AE: adverse event; VRZ: voriconazole;  Concomitant drug: the antibacterial drug used in combination with VRZ;  Count_target AE: number of the target AE reports;  Count_all other AEs: number of all other AE reports related to the drugs recorded in the database;  Neither VRZ nor the concomitant drug: other drugs except for VRZ or the concomitant drug. | | | |  |

| **Supplemental Table 2: Parameters for reporting ratio method analysis** | | |
| --- | --- | --- |
| Drugs | Concomitant drug | Not the concomitant drug |
| VRZ | R_E_ (D_E_/D_b_) | R_1_ (D_1_/D_L_) |
| Not VRZ | R_2_ (D_2_/D_e_) | R_n_ (D_N_/d_e_) |
| Abbreviation：AE: adverse event; VRZ: voriconazole;  R_E_: frequency of the target AE when VRZ combined using with the target drug;  R_1_: frequency of the target AE for VRZ alone;  R_2_: frequency of the target AE for the target drug alone;  R_n_: frequency of the target AE for other drugs except for VRZ or the concomitant drug. | | |

| **Supplemental Table 3: The algorithms, criterion and related parameters of the frequency statistical models referenced in this study** | | | |
| --- | --- | --- | --- |
| Frequency statistical models | Algorithms | Criterion for the DDIs | Meaning of related parameters |
| Reporting Ratio Method ^[12]^ | $R\_\text{diff}=(R_{1}+R_{2})-R_{E}$ | R_diff＞0 | R_1_: frequency of the target AE for voriconazole alone;  R_2_: frequency of the target AE for the target drug alone;  R_E_: frequency of the target AE when voriconazole combined using with the target drug; |
| Ω Shrinkage Measure Model ^[15]^ | $\begin{matrix} E_{E}=D_{E}\times(1-\frac{1}{\max(\frac{R_{n}}{1-R_{n}},\frac{R_{1}}{1-R_{1}})+\max(\frac{R_{n}}{1-R_{n}},\frac{R_{2}}{1-R_{2}})-\frac{R_{n}}{1-R_{n}}+1}) \\ \end{matrix}$ $\Omega=\text{l}og_{2}\frac{D_{E}+0.5}{E_{E}+0.5}$ $\Omega_{0.25}=\Omega\text{-}\frac{\Phi\text{（}0.975\text{）}}{\text{log}_{2}\sqrt{D_{E}}}$    | Ω _0.25_ ＞0 | R_n_: frequency of the target AE for other drugs except for voriconazole or the concomitant drug.  D_E_: the reported number of adverse events when the drugs used in combination  E_E:_the expected number of adverse events when the drugs used in combination.  ϕ(0.975): 97.5% of the standard normal distribution.  Ω_0.25_: the upper limit of bilateral 95% confidence interval of Ω. |
| Combination Risk Ratio Model ^[16]^ | Combination risk ratio=$\frac{PRR_{E}}{\text{m}ax(PRR_{1},PRR_{2})}$ $PRR=\frac{\text{a}/(\text{a}+b)}{c/(c+d)}$ $\text{χ}^{2}=\frac{\text{n}(\vert\text{a}d-bc\vert-\frac{\text{n}}{2})^{2}}{(\text{a}+b)(\text{a}+c)(b+c)(c+d)}$     When calculating PRR_E_ or χ^2^_E_ of drug combination: a=D_E_, b=d_E_, a+b=D_b_, c=D_1_+D_2_+D_N_, d=d_1_+d_2_+d_N_, c+d=D_L_+D_e_+d_e_;  when calculating PRR_1_ or χ^2^_1_ of LZD used alone: a=D_E_+D_1_, b=d_1_+d_E_, a+b=D_L_+D_b_, c=D_2_+D_N_, d=d_2_+d_N_, c+d=De+de;  when calculating PRR2 or χ^2^_2_ of drug2 used alone: a=D_E_+D_2_, b=d_2_+d_E_, a+b=De+D_b_, c=D_1_+D_N_, d=d_1_+d_N_, c+d=D_L_+d_e_ | D_E_≥3, and combination risk ratio > 2, PRR_E_>2, and χ^2^_E_>4 | PRR: proportional reporting ratio.  PRR_1_: PRR of the target AE for voriconazole alone  PRR_2_: PRR of the target AE for the target drug alone  PRR_E_: PRR of the target AE when voriconazole combined using with the target drug;  Meanning of other parameters were shown in Additional fle 1 |
| Chi-Square Statistics Model ^[17]^ | $\text{χ}=\frac{\text{D}_{\text{E}}-E_{E}-0.5}{\sqrt{E_{E}}}$ | χ > 2 | D_E_: the reported number of adverse events when the drugs used in combination  E_E:_ the expected number of adverse events when the drugs used in combination. |
| Abbreviation：AE: adverse event; DDIs: drug–drug interactions. | | | |

| **Supplemental Table 4: Two-by-two contingency table of influencing factors analysis** | | | |
| --- | --- | --- | --- |
| Gender / Age | Target AEs | All other AEs | Total |
| Female / ≤18 years patients | a | b | a+b |
| Male / >18 years patients | c | d | c+d |
| Total | a+c | b+d | n (a+b+c+d) |
| Abbreviation: AEs: adverse events; VRZ: voriconazole;  a: the number of female (or ≤18 years patients) patients with target adverse events. b: the number of female (or ≤18 years patients) patients without target events. c: the number of male patients(or >18 years patients) with target adverse events. d: the number of male patients without target adverse events.  Reporting odds ratio (ROR)=(ad)/(bc) $95\text{\%}CI=e^{I\text{n}\left(\text{ROR}\right)\pm1.96\sqrt{1/\text{a}+1/b+1/c+1/d}}$ | | | |

| **Supplemental Table 5: The other positive** [**safety signals related to DDIs**](https://xueshu.baidu.com/usercenter/paper/show?paperid=84629ec81c1e7c874f18d40068a8991f&site=xueshu_se) **were detected by at least one of the four frequency statistical models when VRZ in combination use with the 38 drugs (n=214)^a^** | | | | | | | | | |
| --- | --- | --- | --- | --- | --- | --- | --- | --- | --- |
| Drugs | Adverse event (PT) | AE reports  (N)^b^ | Reporting rate (%) | | | Reporting ratio method  (R_diff)^c^ | Ω shrinkage measure model  (Ω_0.25_)^d^ | Combination risk ratio model  [CRR(PRR,χ^2^)] | Chi-square statistics model  (χ) |
|  |  |  | VRZ | Target  drug | Both drugs |  |  |  |  |
| **VRZ+Proton pump inhibitors (PPIs)** | | | | | | | | | |
| Aesomeprazole | Pyrexia | 39 | 4.9 | 2.1 | 5.8 | 19.9 | 0.0 | 3.7 (1.7, 116.5) | 1.6 |
| Aesomeprazole | Anaemia | 21 | 1.8 | 1.7 | 3.1 | 12.1 | 0.2 | 3.6 (2.4, 54.3) | 2.1 |
| Aesomeprazole | Hypotension | 19 | 1.7 | 1.1 | 2.8 | 1.4 | 0.4 | 3.1 (2.3, 38.4) | 2.5 |
| Aesomeprazole | Constipation | 13 | 0.8 | 1.5 | 1.9 | 19.0 | 0.0 | 2.1 (2.1, 10.3) | 1.1 |
| Lansoprazole | Pyrexia | 63 | 4.8 | 2.4 | 8.9 | -18.7 | -0.1 | 3.3 (1.7, 148.6) | 1.2 |
| Lansoprazole | Sepsis | 27 | 2.6 | 1.1 | 3.8 | -4.1 | -0.6 | 4.4 (1.3, 94.3) | -0.9 |
| Lansoprazole | Nausea | 54 | 2.4 | 4.6 | 7.6 | -8.3 | -0.5 | 1.3 (1.6, 5.6) | -0.7 |
| Lansoprazole | Dyspnoea | 54 | 2.3 | 3.9 | 7.6 | -19.5 | 0.0 | 1.7 (1.8, 26.8) | 1.7 |
| Lansoprazole | Diarrhoea | 60 | 2.2 | 4.1 | 8.4 | -25.6 | 0.1 | 1.8 (1.9, 33.9) | 2.0 |
| Lansoprazole | Vomiting | 41 | 1.8 | 3.4 | 5.8 | -11.3 | -0.2 | 1.6 (1.6, 14.7) | 0.6 |
| Lansoprazole | Thrombocytopenia | 20 | 1.6 | 0.8 | 2.8 | -13.3 | -0.4 | 3.4 (1.6, 43.5) | 0.2 |
| Lansoprazole | Infection | 18 | 1.5 | 0.8 | 2.5 | -12.2 | -0.3 | 2.5 (1.6, 20.4) | 0.3 |
| Lansoprazole | Cough | 23 | 1.4 | 1.7 | 3.2 | -4.1 | -0.2 | 1.6 (1.8, 7.3) | 0.7 |
| Lansoprazole | Headache | 40 | 1.3 | 3.0 | 5.6 | -22.6 | -0.6 | 1.2 (1.7, 1.7) | -1.2 |
| Lansoprazole | Asthenia | 33 | 1.3 | 2.2 | 4.6 | -23.1 | -0.2 | 1.6 (1.9, 11.1) | 0.8 |
| Lansoprazole | Haemoglobin Decreased | 15 | 0.9 | 0.9 | 2.1 | -16.4 | -0.1 | 2.6 (2.1, 18.5) | 0.9 |
| Lansoprazole | Tremor | 13 | 0.8 | 1.0 | 1.8 | -1.9 | -0.5 | 1.4 (1.7, 1.4) | -0.2 |
| Lansoprazole | Oedema Peripheral | 15 | 0.8 | 1.3 | 2.1 | -4.7 | -0.4 | 2.0 (1.6, 9.9) | 0.2 |
| Omeprazole | Platelet Count Decreased | 33 | 1.7 | 0.8 | 2.8 | -9.8 | 0.0 | 4.7 (1.9, 129.4) | 1.6 |
| Pantoprazole | Anaemia | 47 | 1.7 | 2.6 | 3.5 | 23.8 | -0.2 | 3.6 (2.2, 119.7) | 0.7 |
| Pantoprazole | Hypotension | 42 | 1.7 | 2.1 | 3.2 | 20.6 | 0.0 | 3.1 (2.5, 82.0) | 1.4 |
| Pantoprazole | Haemoglobin Decreased | 22 | 0.8 | 1.4 | 1.7 | 34.3 | -0.4 | 3.1 (2.0, 40.4) | 0.2 |
| Rabeprazole | Pneumonia | 9 | 4.8 | 3.1 | 6.7 | 17.8 | 0.1 | 6.0 (2.5, 49.2) | 1.3 |
| Rabeprazole | Platelet Count Decreased | 4 | 1.8 | 1.0 | 3.0 | -6.2 | -0.1 | 8.1 (3.1, 26.0) | 1.1 |
| Rabeprazole | Decreased Appetite | 4 | 1.1 | 1.9 | 3.0 | 4.0 | -0.1 | 3.8 (3.8, 8.5) | 1.2 |
| **VRZ+Nonsteroidal anti-inflammatory drugs (NSAIDs)** | | | | | | | | | |
| Acetaminophen | Pneumonia | 100 | 4.8 | 2.6 | 5.5 | 35.1 | 0.0 | 4.3 (1.8, 370.6) | 2.3 |
| Acetaminophen | Acute Kidney Injury | 52 | 1.9 | 1.7 | 2.8 | 26.4 | 0.0 | 5.0 (2.3, 228.4) | 1.8 |
| Acetaminophen | Anaemia | 47 | 1.8 | 1.4 | 2.6 | 24.8 | 0.4 | 3.2 (2.3, 101.4) | 3.4 |
| Acetaminophen | Hypotension | 45 | 1.7 | 1.8 | 2.5 | 40.9 | 0.1 | 3.0 (2.3, 84.4) | 2.3 |
| Acetaminophen | Haemoglobin Decreased | 32 | 0.8 | 1.0 | 1.8 | 1.7 | 0.6 | 4.1 (3.4, 100.3) | 3.9 |
| Acetaminophen | Oedema Peripheral | 24 | 0.8 | 1.1 | 1.3 | 41.1 | 0.1 | 2.4 (2.3, 25.9) | 1.7 |
| Aspirin | Pneumonia | 65 | 4.8 | 2.8 | 4.6 | 66.8 | 0.6 | 6.7 (3.1, 444.3) | 5.1 |
| Aspirin | Renal Failure | 28 | 2.0 | 2.0 | 2.0 | 103.4 | 0.1 | 6.1 (2.8, 156.2) | 2.0 |
| Aspirin | Anaemia | 26 | 1.8 | 2.2 | 1.8 | 122.8 | 0.1 | 4.3 (2.4, 87.3) | 1.9 |
| Aspirin | Blood Creatinine Increased | 11 | 1.2 | 0.8 | 0.8 | 151.8 | -0.3 | 5.2 (2.1, 45.4) | 0.4 |
| Celecoxib | Condition Aggravated | 6 | 3.6 | 3.4 | 8.0 | -11.9 | -0.1 | 6.7 (3.0, 35.4) | 1.0 |
| Ibuprofen | Anaemia | 5 | 1.8 | 1.3 | 3.2 | -1.9 | -0.4 | 2.8 (1.9, 5.8) | 0.6 |
| Ibuprofen | Rash | 10 | 1.7 | 3.5 | 6.4 | -19.7 | 0.0 | 2.6 (2.3, 13.1) | 1.2 |
| Ibuprofen | Product Use Issue | 4 | 1.3 | 1.2 | 2.5 | -2.5 | -0.4 | 2.5 (2.2, 3.2) | 0.7 |
| **VRZ+Immunosuppressants** | | | | | | | | | |
| Azathioprine | Pancytopenia | 10 | 1.7 | 1.4 | 3.5 | -8.5 | -0.3 | 13.0 (2.7, 142.5) | 0.5 |
| Cyclophosphamide | Multiple Organ Dysfunction Syndrome | 51 | 1.9 | 0.9 | 3.7 | -23.5 | -0.4 | 23.6 (2.4, 1487.7) | -0.2 |
| Cyclophosphamide | Hypotension | 51 | 1.6 | 1.9 | 3.7 | -4.3 | -0.1 | 2.6 (2.3, 70.0) | 1.2 |
| Cyclophosphamide | Graft Versus Host Disease | 38 | 1.1 | 0.9 | 2.7 | -25.8 | -0.4 | 51.7 (2.9, 2511.2) | -0.2 |
| Cyclophosphamide | Hypoxia | 28 | 0.8 | 0.8 | 2.0 | -21.7 | -0.3 | 8.4 (3.0, 242.8) | 0.2 |
| Cyclophosphamide | Cytomegalovirus Infection | 41 | 0.7 | 1.5 | 2.9 | -25.4 | -0.4 | 25.8 (2.0, 1308.5) | -0.1 |
| Cyclosporine | Pyrexia | 85 | 4.9 | 4.2 | 5.2 | 76.2 | -0.5 | 3.5 (2.0, 214.1) | -1.2 |
| Cyclosporine | Sepsis | 48 | 2.6 | 2.0 | 2.9 | 58.2 | -0.5 | 6.0 (2.1, 270.7) | -1.0 |
| Cyclosporine | Acute Kidney Injury | 35 | 2.0 | 1.1 | 2.1 | 45.7 | -0.2 | 3.5 (2.0, 84.3) | 0.7 |
| Cyclosporine | Pancytopenia | 33 | 1.7 | 1.1 | 2.0 | 41.9 | -0.5 | 8.6 (2.2, 292.9) | -0.5 |
| Cyclosporine | Alanine Aminotransferase Increased | 30 | 1.2 | 1.0 | 1.8 | 20.9 | -0.2 | 6.6 (2.9, 186.4) | 0.8 |
| Cyclosporine | Graft Versus Host Disease | 43 | 1.1 | 1.7 | 2.6 | 7.2 | -0.2 | 80.7 (2.0, 4450.4) | 0.6 |
| Cyclosporine | Aspartate Aminotransferase Increased | 26 | 1.1 | 0.9 | 1.6 | 30.5 | -0.3 | 6.6 (2.7, 160.4) | 0.3 |
| Cyclosporine | Renal Failure Acute | 30 | 1.0 | 1.1 | 1.8 | 12.8 | -0.1 | 7.5 (2.8, 222.8 ) | 1.1 |
| Cytarabine | Septic Shock | 100 | 2.1 | 3.4 | 6.9 | -20.7 | -1.7 | 9.7 (1.3, 1191.0) | -9.9 |
| Cytarabine | Multiple Organ Dysfunction Syndrome | 56 | 1.9 | 1.3 | 3.8 | -18.3 | -1.8 | 10.0 (1.3, 688.0) | -7.7 |
| Cytarabine | Hypokalaemia | 30 | 1.2 | 1.0 | 2.1 | 6.1 | -2.0 | 2.6 (1.1, 44.9) | -6.6 |
| Cytarabine | Blood Bilirubin Increased | 33 | 0.8 | 1.3 | 2.3 | -4.8 | -2.0 | 4.5 (1.1, 134.5) | -6.7 |
| Cytarabine | Hypoxia | 34 | 0.8 | 1.3 | 2.3 | -11.2 | -1.8 | 4.0 (1.2, 111.9) | -6.1 |
| Cytarabine | Acute Respiratory Distress Syndrome | 26 | 0.7 | 1.0 | 1.8 | -7.7 | -2.0 | 5.8 (1.2, 151.5) | -5.9 |
| Dexamethason | Respiratory Failure | 61 | 2.8 | 1.0 | 4.1 | -6.6 | -0.7 | 7.0 (1.5, 388.1) | -1.9 |
| Dexamethason | Septic Shock | 61 | 2.3 | 1.0 | 4.1 | -19.0 | -0.5 | 12.9 (1.9, 826.1) | -1.2 |
| Dexamethason | Product Use Issue | 33 | 1.2 | 0.7 | 2.2 | -12.6 | -0.2 | 1.6 (1.9, 10.1) | 0.8 |
| Dexamethason | Neutrophil Count Decreased | 37 | 0.7 | 0.9 | 2.5 | -31.5 | -0.3 | 8.9 (2.4, 316.1) | 0.4 |
| Hydrocortisone | Pneumonia | 90 | 4.6 | 3.3 | 9.1 | -13.5 | -0.1 | 4.3 (2.0, 333.6) | 1.4 |
| Hydrocortisone | Febrile Neutropenia | 56 | 3.0 | 1.5 | 5.7 | -20.0 | -0.3 | 13.8 (1.9, 903.9) | 0.1 |
| Hydrocortisone | Sepsis | 54 | 2.5 | 1.7 | 5.5 | -23.2 | -0.1 | 7.2 (2.2, 394.7) | 1.1 |
| Hydrocortisone | Acute Kidney Injury | 37 | 1.9 | 1.7 | 3.7 | -4.2 | -0.3 | 3.9 (2.0, 110.9) | 0.2 |
| Hydrocortisone | Renal Failure | 41 | 1.9 | 1.7 | 4.2 | -14.2 | -0.1 | 4.1 (2.3, 132.8) | 1.1 |
| Hydrocortisone | Hypotension | 43 | 1.6 | 2.2 | 4.4 | -11.6 | 0.0 | 3.2 (2.7, 90.8) | 1.6 |
| Methotrexate | Decreased Appetite | 27 | 1.1 | 1.0 | 2.4 | -12.1 | 0.1 | 2.2 (3.0, 26.4) | 1.8 |
| Methylprednisolone | Respiratory Failure | 102 | 2.6 | 2.0 | 6.0 | -24.4 | -0.1 | 12.0 (2.7, 1371.3) | 1.0 |
| Methylprednisolone | Septic Shock | 74 | 2.2 | 1.3 | 4.4 | -20.1 | -0.3 | 16.1 (2.3, 1379.9) | 0.1 |
| Methylprednisolone | Acute Kidney Injury | 57 | 1.9 | 2.1 | 3.4 | 18.5 | -0.6 | 3.6 (2.1, 143.6) | -1.6 |
| Methylprednisolone | Renal Failure | 63 | 1.8 | 2.0 | 3.7 | 2.5 | -0.4 | 3.8 (2.4 , 171.2) | -0.2 |
| Methylprednisolone | Anaemia | 55 | 1.7 | 2.2 | 3.3 | 21.1 | -0.5 | 2.5 (2.0, 67.5) | -0.8 |
| Methylprednisolone | Platelet Count Decreased | 48 | 1.7 | 1.1 | 2.8 | -1.9 | -0.3 | 4.1 (2.0, 150.2) | 0.0 |
| Methylprednisolone | Blood Creatinine Increased | 37 | 1.1 | 1.1 | 2.2 | -1.7 | -0.4 | 4.8 (2.4 , 146.7) | -0.4 |
| Mycophenolate Mofetil | Condition Aggravated | 118 | 3.4 | 2.3 | 6.1 | -5.7 | -0.2 | 3.3 (2.3, 268.3) | 0.6 |
| Mycophenolate Mofetil | Respiratory Failure | 103 | 2.6 | 1.9 | 5.3 | -15.0 | -0.4 | 9.9 (2.6, 1110.6) | -0.7 |
| Mycophenolate Mofetil | Septic Shock | 82 | 2.2 | 1.6 | 4.2 | -10.7 | -0.5 | 14.6 (2.4, 1381.2) | -1.5 |
| Mycophenolate Mofetil | Multiple Organ Dysfunction Syndrome | 81 | 1.8 | 1.3 | 4.2 | -25.5 | -0.3 | 26.8 (2.9, 2653.8) | -0.1 |
| Mycophenolate Mofetil | Graft Versus Host Disease | 74 | 1.0 | 1.2 | 3.8 | -43.2 | 0.0 | 72.2 (2.9, 6781.0) | 2.0 |
| Tacrolimus | Condition Aggravated | 182 | 3.3 | 1.7 | 5.9 | -15.5 | 0.0 | 3.0 (2.4, 354.4) | 2.1 |
| Tacrolimus | Respiratory Failure | 134 | 2.6 | 1.8 | 4.3 | 1.9 | -0.7 | 7.6 (2.2, 1062.6) | -3.7 |
| Tacrolimus | Septic Shock | 110 | 2.2 | 1.5 | 3.6 | 2.7 | -0.8 | 11.5 (2.1, 1449.4) | -3.9 |
| Tacrolimus | Drug Level Increased | 116 | 1.9 | 1.2 | 3.8 | -19.5 | -0.5 | 30.8 (2.6, 4545.0) | -1.8 |
| Tacrolimus | Multiple Organ Dysfunction Syndrome | 118 | 1.7 | 1.2 | 3.8 | -24.6 | -0.4 | 23.0 (2.9, 3376.5) | -0.9 |
| Tacrolimus | Product Use Issue | 66 | 1.1 | 1.0 | 2.1 | -2.2 | -0.1 | 1.6 (2.2, 22.4) | 1.3 |
| Tocilizumab | Pyrexia | 10 | 4.9 | 4.0 | 9.0 | -0.9 | -0.3 | 4.5 (1.8, 40.4) | 0.5 |
| Tocilizumab | Abdominal Pain | 5 | 1.0 | 1.8 | 4.5 | -38.4 | 0.1 | 3.4 (4.3, 10.2) | 1.4 |
| **VRZ+Other antibacterial drugs** | | | | | | | | | |
| Cefazolin | Drug Ineffective | 25 | 10.8 | 4.5 | 22.7 | -32.6 | 0.2 | 2.7 (2.5, 45.9) | 2.1 |
| Cefazolin | Sepsis | 7 | 2.6 | 2.0 | 6.4 | -26.7 | -0.4 | 8.7 (2.9, 55.3) | 0.4 |
| Cefazolin | Septic Shock | 6 | 2.4 | 1.2 | 5.5 | -34.5 | -0.5 | 20.4 (2.7, 125.9) | 0.3 |
| Cefazolin | Pancytopenia | 15 | 1.7 | 1.1 | 13.6 | -79.8 | 1.5 | 38.6 (9.6, 702.6) | 5.9 |
| Cefazolin | Hypokalaemia | 5 | 1.2 | 1.1 | 4.5 | -49.3 | -0.2 | 15.5 (4.4, 73.7) | 0.9 |
| Cefazolin | Pleural Effusion | 5 | 1.2 | 1.2 | 4.5 | -48.1 | -0.1 | 11.0 (4.6, 49.4) | 1.0 |
| Cefazolin | Arthralgia | 6 | 0.8 | 1.4 | 5.5 | -59.7 | -0.2 | 2.2 (4.7, 4.1) | 0.8 |
| Cefepime | Multiple Organ Dysfunction Syndrome | 39 | 1.9 | 1.6 | 4.5 | -20.7 | -0.7 | 24.3 (2.5, 1122.3) | -1.7 |
| Cefepime | Platelet Count Decreased | 34 | 1.7 | 2.2 | 3.9 | 1.1 | -0.9 | 4.5 (2.4, 119.0) | -2.2 |
| Cefepime | Disease Progression | 23 | 1.5 | 0.7 | 2.6 | -17.2 | -0.4 | 3.1 (1.9, 39.9) | 0.0 |
| Cefepime | Pleural Effusion | 28 | 1.1 | 1.7 | 3.2 | -12.3 | -0.8 | 6.0 (2.6, 149.4) | -1.4 |
| Cefepime | White Blood Cell Count Decreased | 24 | 1.1 | 1.8 | 2.8 | 7.4 | -0.9 | 3.2 (2.1, 43.9) | -1.8 |
| Cefepime | Blood Bilirubin Increased | 17 | 0.9 | 0.8 | 2.0 | -14.3 | -0.9 | 8.0 (2.3, 127.6) | -1.2 |
| Cefpodoxime Proxetil | Pneumonia | 5 | 4.8 | 5.8 | 16.1 | -34.3 | -0.3 | 8.6 (4.0, 38.1) | 0.8 |
| Cefpodoxime Proxetil | Nausea | 4 | 2.6 | 3.0 | 12.9 | -56.5 | -0.1 | 2.7 (6.0, 4.7) | 1.1 |
| Ceftazidime | Pancytopenia | 22 | 1.7 | 2.4 | 4.1 | 0.1 | -0.8 | 10.7 (2.9, 265.3) | -1.4 |
| Ceftazidime | Renal Impairment | 24 | 1.4 | 1.7 | 4.5 | -30.5 | -0.2 | 8.0 (4.0, 201.4) | 0.8 |
| Ceftazidime | Multi, Organ Failure | 13 | 1.0 | 1.8 | 2.4 | 16.4 | -1.2 | 14.6 (2.3, 216.8) | -1.8 |
| Ceftriaxone | Off Label Use | 69 | 6.2 | 9.3 | 15.0 | 3.4 | -0.2 | 3.6 (2.1, 199.4) | 0.6 |
| Ceftriaxone | Condition Aggravated | 26 | 3.6 | 2.9 | 5.7 | 15.0 | -0.5 | 3.5 (2.0, 61.9) | -0.4 |
| Ceftriaxone | Respiratory Failure | 23 | 2.8 | 2.3 | 5.0 | 1.8 | -0.6 | 10.5 (2.3, 258.4) | -0.8 |
| Ceftriaxone | Cholestasis | 13 | 1.2 | 1.0 | 2.8 | -22.7 | -0.4 | 24.1 (3.0, 360.2) | 0.2 |
| Cefuroxime Sodium | Pneumonia | 7 | 4.8 | 5.5 | 10.1 | 1.4 | 0.0 | 8.9 (4.1, 62.7) | 1.2 |
| Ciprofloxacin | Pneumonia | 115 | 4.6 | 2.9 | 8.5 | -12.0 | -0.2 | 3.7 (1.9, 313.9) | 0.5 |
| Ciprofloxacin | Febrile Neutropenia | 71 | 3.0 | 1.2 | 5.2 | -19.7 | -0.4 | 11.7 (1.8, 903.9) | -0.6 |
| Ciprofloxacin | Sepsis | 73 | 2.5 | 1.7 | 5.4 | -23.5 | -0.2 | 6.5 (2.3, 446.5) | 0.6 |
| Ciprofloxacin | Neutropenia | 59 | 2.2 | 1.4 | 4.4 | -18.6 | -0.2 | 4.8 (2.1, 234.7) | 0.5 |
| Ciprofloxacin | Product Use In Unapproved Indication | 43 | 2.1 | 0.9 | 3.2 | -5.8 | -0.3 | 2.3 (1.6, 42.8) | 0.0 |
| Clarithromycin | Pneumonia | 21 | 4.8 | 3.6 | 7.7 | 8.4 | -0.4 | 4.1 (2.1, 67.9) | 0.0 |
| Clarithromycin | Respiratory Failure | 11 | 2.9 | 1.1 | 4.0 | -0.6 | -0.6 | 8.8 (1.8, 96.2) | -0.3 |
| Clarithromycin | Acute Kidney Injury | 19 | 1.9 | 3.1 | 7.0 | -27.0 | 0.0 | 8.2 (3.2, 160.1) | 1.3 |
| Clarithromycin | Anaemia | 8 | 1.8 | 1.6 | 2.9 | 16.4 | -0.5 | 2.5 (2.0, 8.3) | 0.2 |
| Clarithromycin | Thrombocytopenia | 7 | 1.6 | 1.3 | 2.6 | 16.0 | -0.8 | 3.9 (2.0, 17.5) | -0.3 |
| Clarithromycin | Renal Impairment | 10 | 1.5 | 1.3 | 3.7 | -25.3 | -0.2 | 7.6 ( 3.2, 71.2) | 0.8 |
| Clarithromycin | Hepatic Function Abnormal | 8 | 1.4 | 0.8 | 2.9 | -22.2 | -0.4 | 13.9 (2.6, 116.1) | 0.3 |
| Clarithromycin | Cough | 10 | 1.4 | 2.4 | 3.7 | 5.4 | -0.3 | 2.3 (2.2, 9.4) | 0.5 |
| Clarithromycin | Electrocardiogram Qt Prolonged | 6 | 0.9 | 1.2 | 2.2 | -2.3 | -0.8 | 10.3 (2.5, 58.0) | -0.3 |
| Imipenem | Pneumonia | 16 | 4.9 | 4.8 | 2.5 | 286.2 | -0.7 | 4.0 ( 2.4, 53.5) | -0.8 |
| Imipenem | Platelet Count Decreased | 9 | 1.8 | 2.7 | 1.4 | 214.1 | -0.7 | 6.8 (3.0, 59.5) | -0.3 |
| Imipenem | Pancytopenia | 5 | 1.8 | 1.9 | 0.8 | 372.8 | -1.5 | 7.2 (2.1, 31.7) | -1.4 |
| Imipenem | Confusional State | 5 | 1.7 | 1.9 | 0.8 | 367.0 | -1.1 | 2.3 (2.1, 4.1) | -0.7 |
| Imipenem | Treatment Failure | 5 | 1.4 | 1.6 | 0.8 | 287.3 | -1.2 | 5.4 (2.6, 20.9) | -0.8 |
| Imipenem | Pleural Effusion | 4 | 1.2 | 1.2 | 0.6 | 278.7 | -1.2 | 5.0 (2.4, 13.7) | -0.6 |
| Imipenem | White Blood Cell Count Decreased | 6 | 1.2 | 1.4 | 0.9 | 171.4 | -0.4 | 4.5 (3.8, 19.8) | 0.4 |
| Imipenem | Blood Creatinine Increased | 4 | 1.2 | 1.2 | 0.6 | 270.8 | -1.2 | 4.6 (2.6, 12.1) | -0.5 |
| Imipenem | Multi, Organ Failure | 7 | 1.0 | 2.6 | 1.1 | 232.4 | -1.0 | 23.8 (2.3, 196.5) | -0.8 |
| Imipenem | Blood Bilirubin Increased | 6 | 0.9 | 0.8 | 0.9 | 85.1 | -0.2 | 16.2 (4.7, 107.0) | 0.8 |
| Imipenem | Haemoglobin Decreased | 5 | 0.9 | 2.0 | 0.8 | 274.6 | -1.0 | 3.7 (2.2, 11.3) | -0.5 |
| Imipenem And Cilastatin Sodium | Pneumonia | 16 | 4.8 | 4.1 | 9.4 | -5.4 | -0.6 | 4.0 (2.4, 20.4) | -0.3 |
| Imipenem And Cilastatin Sodium | Platelet Count Decreased | 9 | 1.7 | 2.3 | 5.3 | -23.7 | -0.5 | 6.8 (3.0, 8.5) | 0.0 |
| Imipenem And Cilastatin Sodium | Pancytopenia | 5 | 1.7 | 1.7 | 2.9 | 15.9 | -1.4 | 7.2 (2.1, 1.0) | -1.1 |
| Imipenem And Cilastatin Sodium | Confusional State | 5 | 1.7 | 1.7 | 2.9 | 14.4 | -1.0 | 2.3 (2.1, 3.1) | -0.4 |
| Imipenem And Cilastatin Sodium | Treatment Failure | 5 | 1.4 | 1.4 | 2.9 | -5.3 | -1.0 | 5.4 (2.6, 1.3) | -0.5 |
| Imipenem And Cilastatin Sodium | Pleural Effusion | 4 | 1.2 | 1.0 | 2.4 | -6.8 | -1.1 | 5.0 (2.4, 6.0) | -0.4 |
| Imipenem And Cilastatin Sodium | White Blood Cell Count Decreased | 6 | 1.1 | 1.2 | 3.5 | -33.6 | -0.2 | 4.5 (3.8, 0.1) | 0.7 |
| Imipenem And Cilastatin Sodium | Blood Creatinine Increased | 4 | 1.1 | 1.0 | 2.4 | -7.5 | -1.1 | 4.6 (2.6, 5.9) | -0.4 |
| Imipenem And Cilastatin Sodium | Multi, Organ Failure | 7 | 1.0 | 2.3 | 4.1 | -19.9 | -0.9 | 23.8 (2.3, 15.0) | -0.5 |
| Imipenem And Cilastatin Sodium | Blood Bilirubin Increased | 6 | 0.9 | 0.7 | 3.5 | -54.3 | -0.1 | 16.3 (4.7, 1.7) | 1.0 |
| Imipenem And Cilastatin Sodium | Haemoglobin Decreased | 5 | 0.9 | 1.8 | 2.9 | -10.2 | -0.8 | 3.7 (2.1, 2.0) | -0.1 |
| Levofloxacin | Platelet Count Decreased | 38 | 1.7 | 1.3 | 2.6 | 15.3 | -0.4 | 4.3 (2.1, 124.5) | -0.3 |
| Levofloxacin | Renal Impairment | 33 | 1.4 | 0.9 | 2.2 | 6.0 | -0.3 | 4.8 (2.2, 128.6) | 0.1 |
| Levofloxacin | Hypokalaemia | 29 | 1.2 | 0.9 | 2.0 | 4.3 | -0.5 | 7.5 (2.3, 209.3) | -0.3 |
| Levofloxacin | White Blood Cell Count Decreased | 35 | 1.1 | 1.1 | 2.4 | -6.5 | 0.0 | 3.9 (3.1, 97.8) | 1.6 |
| Linezolid | Drug Ineffective | 170 | 10.9 | 5.3 | 18.3 | -11.3 | -0.4 | 1.5 (1.6, 57.0) | -1.6 |
| Linezolid | Febrile Neutropenia | 39 | 3.1 | 0.6 | 4.2 | -11.6 | -1.0 | 7.3 (1.3, 262.8) | -2.6 |
| Linezolid | Respiratory Failure | 60 | 2.7 | 2.1 | 6.4 | -25.2 | -0.7 | 9.1 (2.2, 539.6) | -1.9 |
| Linezolid | Septic Shock | 73 | 2.1 | 2.0 | 7.8 | -47.4 | -0.2 | 20.3 (3.4, 1680.4) | 0.6 |
| Linezolid | Multiple Organ Dysfunction Syndrome | 60 | 1.8 | 1.1 | 6.4 | -54.0 | -0.1 | 31.0 (3.2, 2161.9) | 1.4 |
| Linezolid | Visual Impairment | 25 | 1.8 | 0.6 | 2.7 | -10.6 | -0.7 | 2.5 (1.4, 27.1) | -1.2 |
| Linezolid | Headache | 35 | 1.4 | 1.4 | 3.8 | -27.9 | -1.4 | 0.6 (2.6, 8.2) | -4.2 |
| Linezolid | Product Use Issue | 31 | 1.2 | 1.1 | 3.3 | -30.0 | 0.1 | 2.0 (2.6, 20.2) | 1.8 |
| Linezolid | Hypokalaemia | 38 | 1.1 | 1.0 | 4.1 | -49.2 | -0.1 | 9.6 (3.4, 363.0) | 1.0 |
| Linezolid | Pleural Effusion | 23 | 1.1 | 0.6 | 2.5 | -27.7 | -0.5 | 4.2 (2.0, 66.6) | -0.4 |
| Linezolid | Decreased Appetite | 27 | 1.1 | 1.5 | 2.9 | -11.5 | -0.4 | 1.4 (1.9, 3.9) | 0.0 |
| Linezolid | Abdominal Pain | 20 | 1.0 | 1.1 | 2.1 | -1.0 | -0.8 | 1.0 (1.8, 0.0) | -1.2 |
| Linezolid | Constipation | 34 | 0.7 | 0.5 | 3.7 | -65.8 | 0.2 | 1.9 (4.8, 19.0) | 2.4 |
| Linezolid | Pathogen Resistance | 21 | 0.7 | 1.2 | 2.3 | -15.9 | -1.1 | 29.5 ( 1.8, 694.8) | -2.1 |
| Meropenem | Febrile Neutropenia | 118 | 2.8 | 3.3 | 5.8 | 6.3 | -0.9 | 12.1(2.6, 1741.8) | -4.7 |
| Meropenem | Respiratory Failure | 146 | 2.4 | 3.3 | 7.2 | -19.4 | -0.5 | 12.1 (3.3, 2155.9) | -1.6 |
| Meropenem | Septic Shock | 132 | 2.0 | 4.4 | 6.5 | -1.5 | -0.8 | 20.2 (2.2, 3467.2) | -4.4 |
| Meropenem | Multiple Organ Dysfunction Syndrome | 136 | 1.5 | 3.3 | 6.7 | -27.8 | -0.4 | 38.6 (3.0, 7131.1) | -1.0 |
| Meropenem | Renal Impairment | 61 | 1.3 | 2.1 | 3.0 | 13.8 | -0.9 | 4.8 (2.2, 261.2) | -3.2 |
| Meropenem | Hypoxia | 46 | 0.8 | 1.2 | 2.3 | -14.8 | -0.6 | 8.5 (3.0, 428.8) | -1.2 |
| Meropenem | Cardiac Failure | 28 | 0.8 | 1.0 | 1.4 | 30.0 | -0.9 | 2.2 (2.1, 24.8) | -1.9 |
| Sulfamethoxazole | Pancytopenia | 53 | 1.8 | 2.1 | 1.7 | 132.6 | -0.8 | 9.5 (2.2, 515.8) | -2.4 |
| Sulfamethoxazole | Alanine Aminotransferase Increased | 39 | 1.2 | 1.1 | 1.2 | 89.1 | -0.4 | 5.9 (2.7, 200.0) | -0.2 |
| Sulfamethoxazole | Aspartate Aminotransferase Increased | 34 | 1.2 | 1.0 | 1.1 | 100.8 | -0.5 | 5.9 (2.4, 174.8) | -0.6 |
| Vancomycin | Condition Aggravated | 154 | 3.3 | 2.5 | 6.6 | -12.7 | -0.3 | 3.0 (2.3, 306.3) | -0.3 |
| Vancomycin | Febrile Neutropenia | 119 | 2.9 | 2.1 | 5.1 | -2.0 | -0.9 | 10.0 (2.0, 1332.9) | -4.5 |
| Vancomycin | Respiratory Failure | 124 | 2.6 | 2.5 | 5.3 | -5.3 | -0.8 | 8.4 (2.3, 1120.1) | -4.0 |
| Vancomycin | Neutropenia | 108 | 2.1 | 2.4 | 4.6 | -5.0 | -0.7 | 4.5 (2.3, 412.0) | -2.7 |
| Vancomycin | Multiple Organ Dysfunction Syndrome | 98 | 1.8 | 1.5 | 4.2 | -23.0 | -0.6 | 22.7 (2.7, 2780.2) | -2.1 |
| Vancomycin | White Blood Cell Count Decreased | 49 | 1.1 | 1.1 | 2.1 | 4.4 | -0.6 | 2.4 (2.2, 54.3) | -1.3 |
| Vancomycin | Hypoxia | 52 | 0.7 | 1.2 | 2.2 | -13.7 | -0.7 | 7.8 (2.2, 420.4) | -1.9 |
| Abbreviation: AE: adverse event; CRR: Combination risk ratio; DDIs: drug-drug interactions; DE: the reported number of adverse events when the drugs used in combination; N: Number; PRR: proportional reporting ratio; PT: preferred term; VRZ: Voriconazole.  ^a^: Criterion for positive safety signals related to DDIs of the four frequency statistical models: R_diff < 0; Ω_0.25_＞0; CRR＞0, PRR＞2, χ^2^＞4; χ＞2.  ^b^: The reported number of target AEs when two drugs are used together, and its meaning equivalent to D_E_ shown in Additional file 1;  ^c^: R_diff: the observed AE frequency of combined drug use (R_E_) was greater than the expected AE frequency (the sum of the occurrence frequency when each drug used separately).  ^d^:Ω_0.25_: the upper limit of bilateral 95% confidence interval of Ω. | | | | | | | | | |
